# Solvatomorphism of Moxidectin

**DOI:** 10.3390/molecules26164869

**Published:** 2021-08-11

**Authors:** Toni Grell, Mauro Barbero, Franco Pattarino, Giovanni Battista Giovenzana, Valentina Colombo

**Affiliations:** 1 Dipartimento di Chimica, Università degli Studi di Milano, Via Golgi 19, I-20133 Milano, Italy; toni.grell@unimi.it; 2 Dipartimento di Scienze del Farmaco, Università del Piemonte Orientale A. Avogadro, Largo Donegani 2/3, I-28100 Novara, Italy; mauro.barbero@uniupo.it (M.B.); franco.pattarino@uniupo.it (F.P.); giovannibattista.giovenzana@uniupo.it (G.B.G.)

**Keywords:** moxidectin, solvatomorphism, X-ray diffraction, variable-temperature PXRD

## Abstract

The solvatomorphism of the anthelmintic drug moxidectin is investigated, and a new solvatomorph with nitromethane is reported. Moreover, the hitherto unknown crystal structures of the solvatomorphs with ethanol and 2-propanol are reported and discussed. The thermal characterization of these solvatomorphs through variable-temperature powder X-ray diffraction analysis (VT-PXRD) is also described, providing new insights into the crystallochemistry of this active pharmaceutical ingredient.

## 1. Introduction

Polymorphism and solvatomorphism are highly important phenomena in the pharmaceutical industry [1,2]. Different arrangements of the molecules of an active pharmaceutical ingredient (API) in the solid-state give crystal structures with properties which have impact on the manufacturing process (API melting temperature, hygroscopicity and compactability), pharmaceutical behavior (solubility and dissolution rate of the API) and shelf life (stability) of medicinal products. As a consequence, the crystal form of the API may significantly affect the efficacy and safety of a medicinal product [3]. Therefore, the identification of the solid-state forms of an API and the investigation of their properties is of paramount importance for the marketing authorization of the medicine. The well-known case of the antiviral drug ritonavir [4] illustrates this relevance and the reasons why nowadays the presence of polymorphs and solvatomorphs are thoroughly studied. In contrast, the detailed investigation of their solid-state structures is often paid little attention even though this can reveal further insights that might allow for a deeper understanding and potentially even predictions [5].

**Scheme 1:**
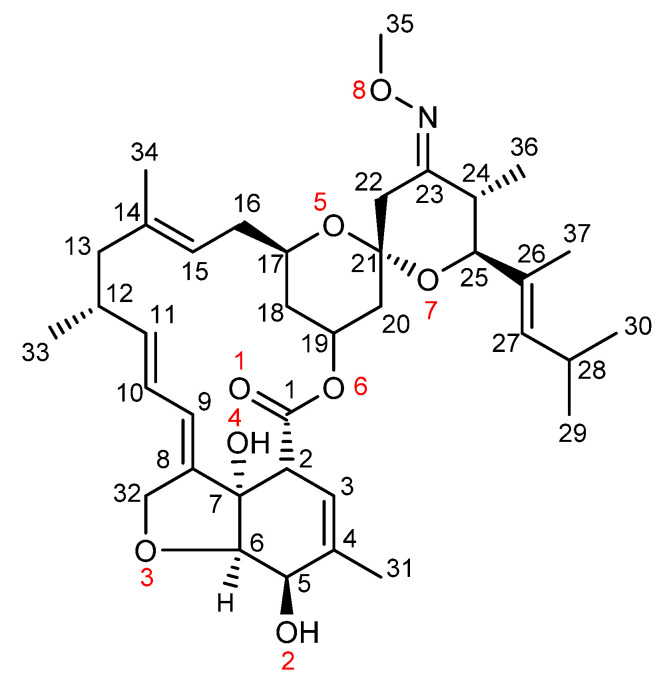
Structural formula of moxidectin including the labels used in this paper for SC-XRD structure description (numbers in black for carbon and red for oxygen atoms).

This applies to moxidectin (hereafter Moxi, Scheme 1), a bioactive substance used in long-acting formulations such as tablet, oral drench, oral gel/paste, injectable, pour-on and topical solution, in the prevention of heartworm in dogs (e.g., ProHearth^®^) and for the treatment of internal and external parasites in cattle, sheep, horses and deer (e.g., Cydectin^®^, Quest^®^, Equest^®^) [6,7,8,9]. Moxidectin has recently been approved by the FDA for the treatment of river blindness [10] and is nowadays under investigation for pest control on crops. The molecular structure of moxidectin is based on a 16-membered macrocyclic lactone ring, a feature shared with the other members of the milbemycin group, as fermentation products of *Streptomyces* [11].

While the overwhelming part of the research on moxidectin is focused on its biological activity, preparation and formulation, investigations on the poly- or solvatomorphism are scarce. To date, one polymorph of the pure form [12] (usually referred to as Form I), an amorphous form and three solvatomorphs with ethanol (EtOH), 2-propanol (*i*PrOH) and 1-butanol (*n*BuOH) have been reported [13]. However, structural data only exist for the pure Form I (summarized in Table 1). This is not unexpected for pharmaceutical compounds, for which the complete structural information is often lacking in the scientific literature and in the patent landscape, mostly because of the difficulty in growing single crystals of metastable phases from solution (thus preventing structure determination by conventional X-ray diffraction methods).

Our research group is interested in the characterization of polymorphic drugs by single-crystal XRD [14] and, when single-crystals are not available, with ab initio methods applied to laboratory-scale powder X-ray diffraction data (PXRD), collected from microcrystalline powders [15,16]. Regarding solid moxidectin, the scientific literature and the patents deposited in the last years, witness the occurrence of different crystal forms, for which very limited structural knowledge is available. Indeed, given the lack of structural information on this drug, we have undertaken a systematic screening for crystallization solvents and conditions, with the aim of reaching a deeper knowledge of its crystal chemistry. Table 1 reports a synoptic collection of the crystal forms described in the literature and patents, while this paper reports on the understanding of the solid-state forms of moxidectin, with the comprehensive structural characterization of its solvatomorphs and the discovery of a novel one.

## 2. Results and Discussion

### 2.1. Preliminary Considerations on Moxidectin Reports

**Figure 1:**
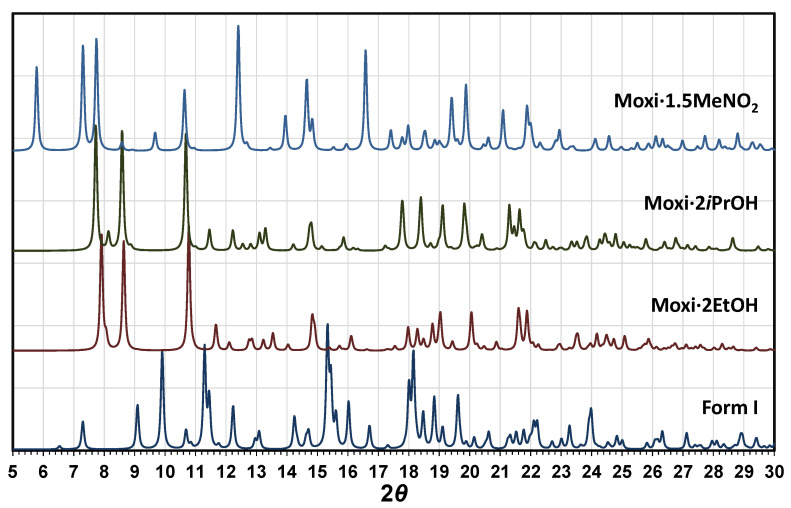
Calculated PXRD patterns (radiation Cu-Kα) for moxidectin known crystal forms: bottom to top: Form I; Moxi·2EtOH; Moxi·2*i*PrOH; Moxi·1.5MeNO_2_. Patterns were calculated from single crystal XRD using the Mercury (CCDC) powder diffraction simulation tool.

As already anticipated in the introduction, despite the recent FDA approval, very few reports have been published on the solid-state chemistry of moxidectin [12,13,17,18]. Crystalline form I was identified in 1988 and its structure revealed by single-crystal X-ray diffraction analysis (deposited in the CSD [19] with reference code GETBOW [12]). The calculated powder diffraction pattern is reported in Figure 1. Later, in 2013, a patent [13] was published which reports on the crystallization of the amorphous form of moxidectin into Form I, exploiting heating/cooling procedures in an oil bath. Moreover, the existence of three moxidectin solvates was stated, namely with ethanol, 2-propanol and 1-butanol. In this patent, these solvates are compared by DSC (Differential Scanning Calorimetry) analysis and visual comparison of the PXRD patterns. In addition to that, a graphical representation of the crystal structure, together with crystallographic space group, and unit cell metrics is reported for the ethanol solvate. However, this crystal structure has never been deposited and thus the quality and origin of the crystallographic data remains doubtful. More recently, two additional works appeared on the crystal growth of the moxidectin-ethanol solvate. In 2015, Shi et al. described the solid-state transformation of the ethanol solvate to the amorphous form, indicating that the ethanol solvate may be not stable during drying, storage and transport of the API or of solid pharmaceutical forms [18]. Moreover, in the same paper, the reversible transformation between the ethanol solvate and form I in water/ethanol mixtures was reported. Two years later, the same group studied the mechanism of formation of a gel and its subsequent transformation to form I [17]. Moxidectin shows indeed a promising rich crystal chemistry, which needs to be clarified and accurately described. Here we intend to give a comprehensive characterization of the known crystal forms of moxidectin as well as a new one, reporting detailed structural data, as a benchmark on its solid-state chemistry for the stakeholders dealing with polymorphism and solvatomorphism of this drug.

### 2.2. Ethanol, 2-Propanol, Methanol and 1-Butanol Solvates

Systematic crystallization trials have been performed to verify the existence of the already claimed solvatomorphs and furthermore explore yet unknown ones. From the respective solvents we could grow single crystals suitable for SC-XRD experiments for the solvatomorphs containing ethanol and 2-propanol. All our attempts to crystallize the 1-butanol solvatomorph instead failed and yielded only powders of the amorphous form, which showed the typical amorphous trace when analyzed with PXRD. Varying crystallization conditions and solvents, we furthermore discovered a hitherto unknown solvatomorph containing nitromethane (Section 2.3).

**Figure 2:**
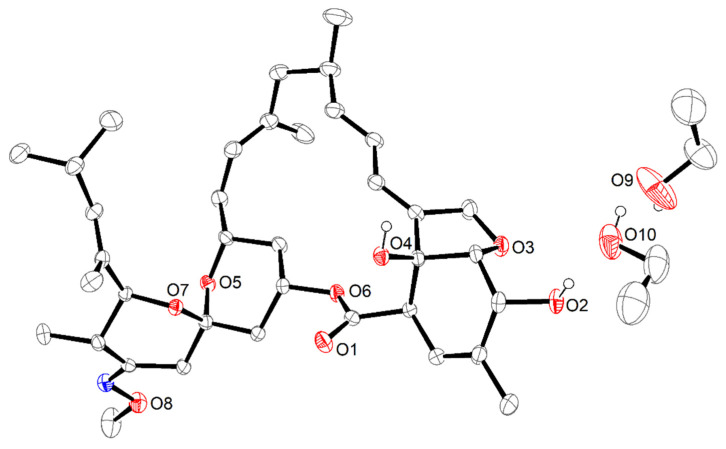
Asymmetric unit in the crystal structure of the ethanol solvate (Moxi·2EtOH) containing the molecular structures of moxidectin and two independent ethanol molecules shown with anisotropic displacement parameters (ADPs). ADPs are shown with 50% probability. Color code: C, grey; N, blue; O, red; H, white.

**Figure 3:**
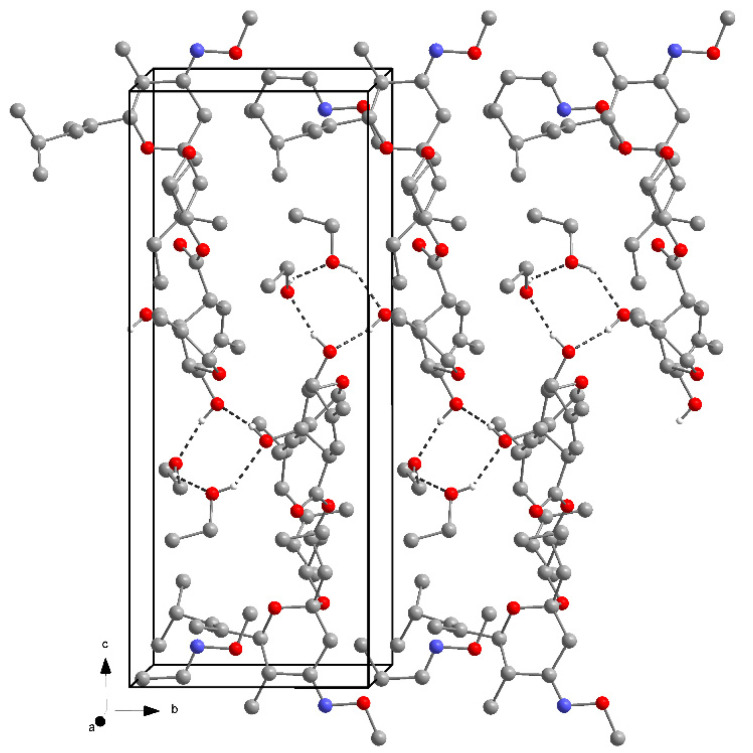
Representation of the chains along the crystallographic [010] direction formed by hydrogen bonds between the molecules in Moxi·2ROH (R = Et, *i*Pr; exemplarily shown for the EtOH solvate). Color code: C, grey; N, blue; O, red; H, white.

**Figure 4:**
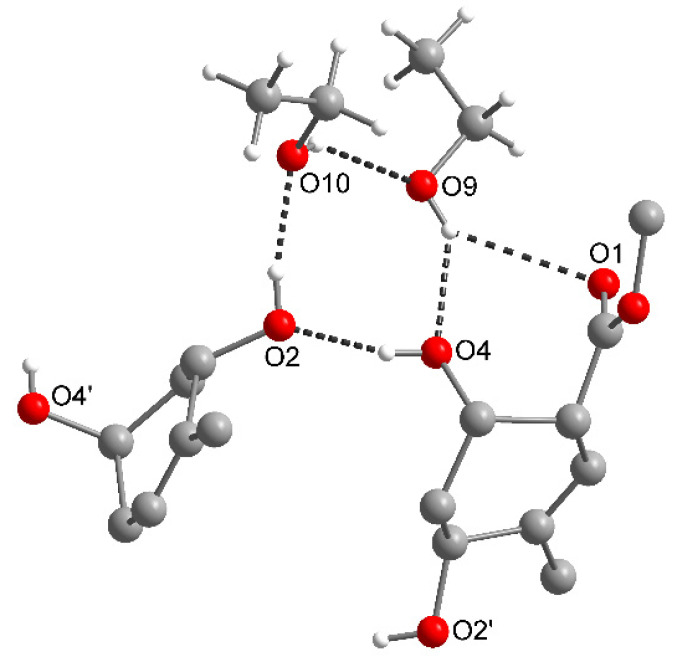
Four-membered cyclic hydrogen bond motif between two moxidectin molecules (only fragments shown) and two ethanol solvent molecules. Selected distances O1–O9 3.255(4) Å, O2–O4 2.760(2) Å, O2–O10 2.700(3) Å, O4–O9 2.782(4) Å, O9–O10 2.719(5) Å. Colour code: C, grey; N, blue; O, red; H, white.

Results from SC-XRD analyses showed that the crystal structures of the ethanol and 2-propanol solvatomorphs are isostructural. The compounds crystallize in the space group *P*2_1_ with one moxidectin and two solvent molecules in the asymmetric unit (here reported as Moxi·2EtOH, Moxi·2*i*PrOH), all lying in general position (Figure 2). Each moxidectin molecule is connected to two neighboring ones through intermolecular hydrogen bonds (HBs) between the hydroxyl groups of the hexahydrobenzofuran ring (O2 and O4, see Scheme 1 for numbering scheme). These HBs contacts (O4–H···O2, 2.760(2) Å) generate an alignment of the moxidectin molecules creating chains along the crystallographic [010] direction (Figure 3). Interestingly, the two independent solvent molecules are paired by hydrogen bonds and furthermore connected to the moxidectin molecules, thus forming a four-membered cyclic HB pattern containing two solvent (ROH) and two moxidectin molecules (Figure 4). This cyclic arrangement is based on intermolecular hydrogen bonds of the type O–H···O, which range between 2.700(3) and 2.782(4) Å. The fact that the hydrogen atom of the hydroxyl group of one moxidectin molecules (O4) is engaged in this HB pattern, and that the OH group of the involved ethanol molecule (O9) forms a bifurcated HB interacting with a second oxygen (O1) of the same moxidectin molecule (Figure 4), leads to the absence of the *intra*molecular hydrogen bond, which was observed in form I, Moxi·1.5MeNO_2_ (see below) and in other milbemycins [20]. It should be noted here that the presence of many electronegative oxygen atoms that can behave as HB acceptors, may generate weak HBs of the type C–H···O that help stabilizing the structure. As for form I, one such interaction is also observed in these structures with distances of 3.377(3) Å (Moxi·2EtOH) and 3.429(3) Å (Moxi·2*i*PrOH) (see discussion below and Section S3 in the Supplementary Material).

In addition to the crystal structures of Moxi·2EtOH and Moxi·2*i*PrOH, observations on the crystallization behavior of moxidectin in other alcohols, namely methanol (MeOH) and 1-butanol (*n*BuOH), have been reported in the previously cited patent [13]. The authors describe that crystallization from methanol solutions exclusively gives rise to form I. Most likely the methanol molecule is too small to stabilize the formation of the isostructural solvatomorph, as in the case of bulkier EtOH and *i*PrOH alcohols. Beyond that, the same patent claims the existence of a 1-butanol solvatomorph, containing 1.5 solvent molecules per moxidectin unit. Unfortunately, we failed to reproduce a sample of this 1-butanol solvatomorph, instead obtaining merely amorphous powders. However, given our findings on the isostructural nature of ethanol and 2-propanol solvates, we considered the possibility that this also applies to the chemically similar, yet bulkier, *n*BuOH. We assumed that *n*BuOH would stabilize the isomorphous crystal structure containing 2 instead of 1.5 solvent molecules, as previously claimed. In fact, the authors based this conclusion on DSC and TGA measurements, reporting that the *n*BuOH solvated sample was air-dried prior to analysis. This treatment could thus have induced a partial desolvation of the clathrated solvent molecules [13]. This effect was already observed for the ethanol solvatomorph, where a slow loss of ethanol molecules has been reported at relatively low temperatures (303.15 K) [18]. We confirm the same observation, as Moxi·2EtOH single crystals progressively lose their crystallinity during a room temperature overnight XRD data collection, owed to slow solvent loss. Indeed, the loss of some solvent molecules from the bulk of the *n*BuOH solvatomorph, at room temperature, is plausible. To support this assumption, quantum mechanical calculations (details in Section 3.4 and Section S3 in the Supplementary Material) were carried out. We performed a geometry optimization on the hypothetical isostructural solvatomorph Moxi·2*n*BuOH using DFT calculations. For that purpose, we modified the crystal structure of the ethanol solvatomorphs by replacing hydrogen atoms in the solvent molecules by ethyl groups. This input was subsequently optimized, and a structure was obtained which was practically identical to the given input and isomorphous to the structures of ethanol and 2-propanol. This shows that the claimed Moxi·2*n*BuOH structure is theoretically possible and that also *n*BuOH could stabilize its formation. Nevertheless, further investigations and crystal growth experiments are indispensable for the understanding of the crystallization kinetics as well as dynamics in alcohols. Furthermore, powder or SC-XRD analysis will be essential to clarify and confirm the identity of the *n*BuOH form.

### 2.3. The Nitromethane Solvatomorph, Moxi·1.5MeNO_2_

By keeping a nitromethane solution of moxidectin at 4 °C, crystals of a new solvatomorph have been obtained. The SC-XRD analysis revealed that in this case moxidectin has crystallized in the space group *P*2_1_2_1_2 with a unit cell containing four moxidectin molecules as well as 6 nitromethane molecules, corresponding to the chemical formula Moxi·1.5MeNO_2_.

**Figure 5:**
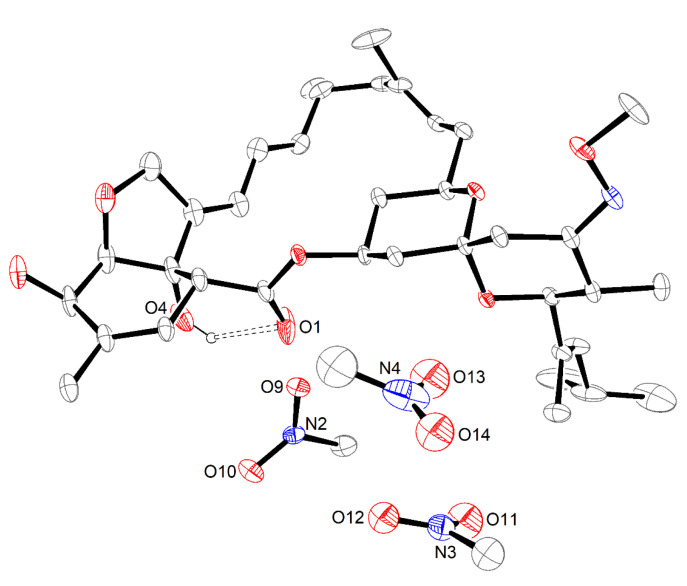
Asymmetric unit of the crystal structure of the nitromethane solvate Moxi·1.5MeNO_2_ showing a one moxidectin and three nitromethane molecules. For the disordered nitromethane molecules only one position is shown. Anisotropic displacement parameters are shown with 30% probability.

**Figure 6:**
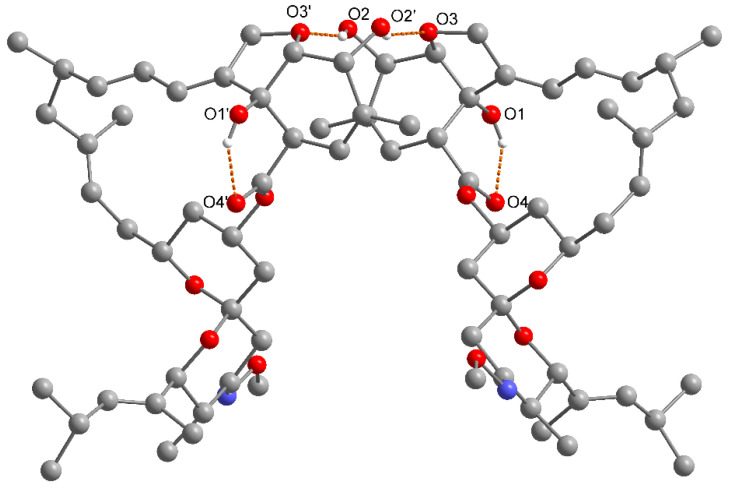
Crystal structure of Moxi·1.5MeNO_2_ showing a dimer of two moxidectin molecules. Selected distances O2–O3′ 2.863(5) Å, O1–O4 2.759(6) Å. Color code: C, grey; N, blue; O, red; H, white.

In the crystal structure of Moxi·1.5MeNO_2_**,** groups of three nitromethane molecules are located in a cavity and disordered around the twofold axis (Figure 5, in the crystal structure an overlap of the two positions is observed). In contrast to the already described alcoholate solvates, there are no strong HBs between the solvent molecules and the moxidectin. Instead, two *inter*molecular HBs are observed between adjacent moxidectin molecules (O2–H···O3, 2.869(4) Å) forming a dimer located on the twofold axis (Figure 6). In addition, the same *intra*molecular HB observed in crystal form I is present here (O4–H53···O1, 2.763(5)). Interestingly, six weak HBs of the type C–H···O can be observed, with oxygen being acceptor atom in both moxidectin and the solvent molecules. The distances observed for these contacts fall in the range of 3.254(6)-3.58(1) Å. This is much more compared to the solvatomorphs with ethanol and 2-propanol, where only one of these interactions is observed.

### 2.4. Comparative Analysis of Moxidectin Crystal Forms

**Figure 7:**
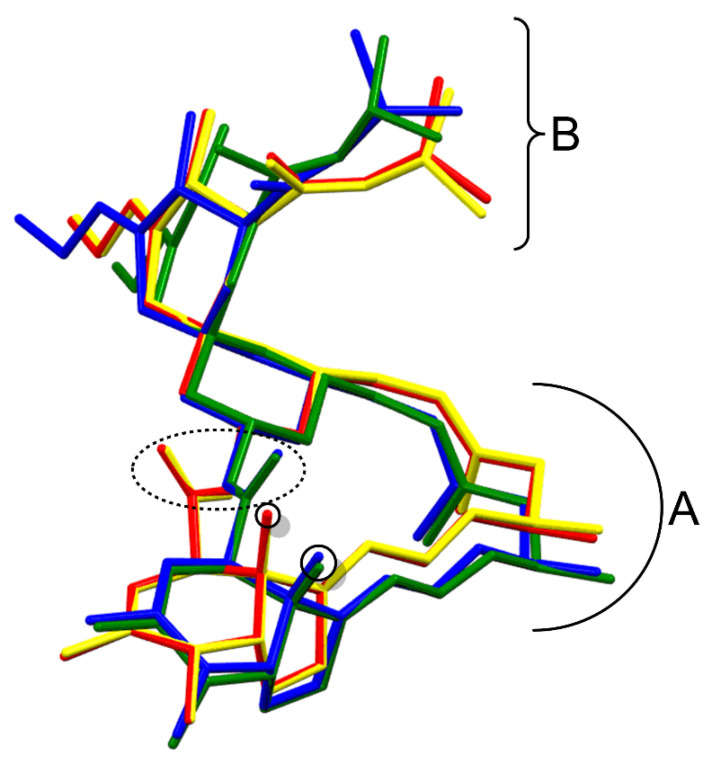
Comparison of the conformations of the moxidectin molecule in the crystal forms described in this paper. The graphical representation shows the different conformations of moxidectin solvatomorphs. Color code: yellow (Moxi·2EtOH), red (Moxi·2*i*PrOH), green (Moxi·1.5MeNO_2_) and blue (form I, CSD reference code GETBOW [12]). The orientation of the 16-membered ring (A) and the spirocycle (B) are indicated.

A deeper understanding of the *inter*- and *intra*molecular interactions in the crystal structures of the solvatomorphs can be gained by inspecting their molecular structures. Figure 7 shows a comparison between all conformations of the moxidectin molecules in the different solvatomorphs. The conformation of the macrocycle in the different forms is generally conserved, as expected from the many structural features contributing to the reduction of its conformational freedom, represented by: (i) the ring fusion with the bicyclic hexahydrobenzofuran system, (ii) the ring fusion with the spirobipyran system, (iii) the endocyclic olefinic C=C bonds (two of them conjugated). Nevertheless, even if small, significant differences can be found. Within the four crystal structures, two conformations can be distinguished: the first is found in the alcoholate solvatomorphs (Moxi·2ROH, R = Et, *i*Pr), whose conformations (depicted in yellow and red in Figure 7) are practically identical, further underlining their isomorphism. A different conformation is found in form I and Moxi·1.5MeNO_2_ (depicted in blue and green in Figure 7). Here, besides a negligible variation of the torsion angles of the terminal groups (C26–C30), a different orientation of the lactone group C1(=O1)–O6 (highlighted with dotted circle) is clearly observed. Moreover, in form I and Moxi·1.5MeNO_2_, the exocyclic lactone carbonyl group C1=O1 points inwards the macrocycle, thereby reducing its distance to the hexahydrobenzofuran ring. In this way, the previously described *intra*molecular HB with the respective hydroxyl group (O4–H53···O1, solid circles in Figure 7), can be established. In contrast to that, in the alcohol-based solvatomorphs (Moxi·2ROH, R = Et, *i*Pr) more attractive *inter*molecular interactions are possible as suitable HB-donor solvent molecules are present: the hydroxyl group O4–H53 here is engaged in the HB pattern described above (Figure 4), not allowing the formation of the *intra*molecular HB with O1. To achieve this, the carbonyl group C1=O1 alters its torsion to point outwards from the macrocycle, consequently inducing a change in the orientation of the hexahydrobenzofuran ring. These effects sum up to have noticeable influence on the mutual orientation of the 16-membered ring (labeled A) and the spirocycle (labeled B) as visualized in Figure 7, leading to a significant increase in the puckering of the moxidectin molecule, with the latter showing a more pronounced concavity.

These considerations demonstrate the substantial relationship between the conformation and the crystal structure of the moxidectin solvatomorphs: in the presence of suitable protic solvent molecules, a HB network is established between the two co-formers, stabilizing the crystal structure (Moxi·2ROH, R = Et, *i*Pr). Instead, in the absence of protic solvent molecules fewer strong HBs are established, and their absence is compensated by a larger number of weak intermolecular interactions of the type C–H···O (form I, Moxi·1.5MeNO_2_), contributing to the stabilization of the crystal structure. A list of all HB interactions is given in Table S1 (Supplementary Material).

### 2.5. Variable-Temperature Powder X-ray Diffraction (VT-XRPD) Analysis

**Figure 8:**
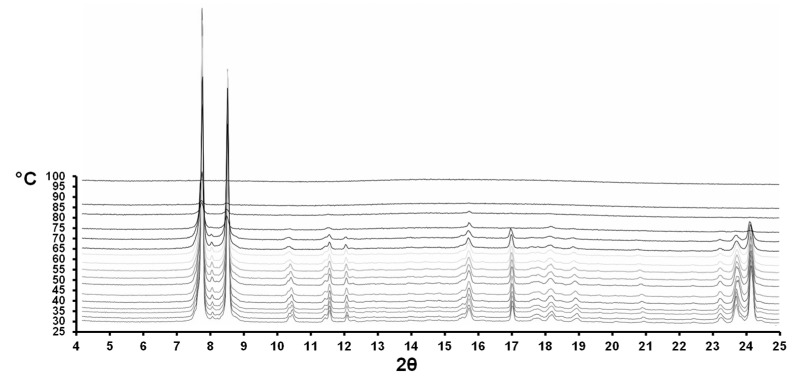
Two-dimensional contour plot as a function of 2θ and temperature for the collection of PXRD patterns measured at elevating temperatures in the range of 25–100 °C for Moxi·2*i*PrOH. The collection of PXRD patterns shows that, by raising the temperature, the sample loses crystallinity at about 70 °C, and becomes amorphous. No peak shifts are observed. As a sign of amorphization, the lowering of peak intensities, starting from 65 °C, is observed. The sample can be considered fully amorphous at about 85 °C.

**Figure 9:**
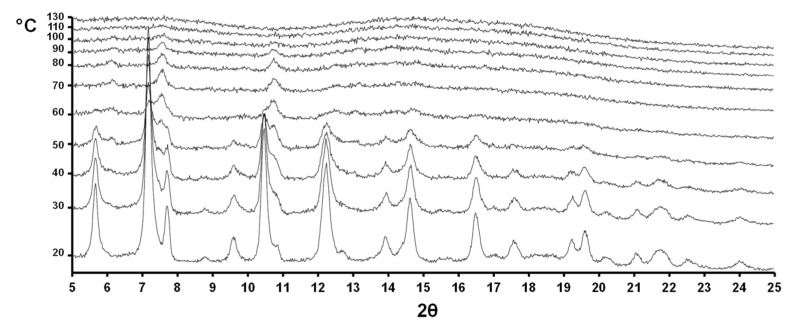
Two-dimensional contour plot as a function of 2θ and temperature for the collection of PXRD patterns measured at elevating temperatures in the range of 25–130 °C for Moxi·1.5MeNO_2_. The collection of PXRD patterns shows that the sample loses crystallinity at about 50 °C and becomes amorphous by raising the temperature. No peak shifts are observed. As a sign of amorphization, the lowering of peak intensities, starting from 50 °C, is observed. The sample can be considered fully amorphous at about 100 °C.

In order to answer the question whether the observed solvatomorphs discussed in this work show possible additional phases or transitions, we investigated them using variable-temperature powder X-ray diffraction (VT-PXRD) analysis. This analytical tool is very useful for pharmaceutical compounds [15,16,21] or coordination polymers [22,23]. While classical thermal analyses can detect solid-state phenomena without giving detailed information about the crystallinity of the species under investigations, VT-PXRD has the power to detect all *irreversible* and *reversible* phenomena which occur at specific temperatures, giving vital information on the crystal chemistry of the investigated samples. The observable phenomena are numerous, and can be related, for example, to a solid-solid phase transition; the loss of solvent molecules with subsequent formation of new (amorphous or crystalline) phase; melting and subsequent amorphization or recrystallization toward a new crystal phase, etc. For moxidectin, we performed experiments on Moxi·2*i*PrOH and Moxi·1.5MeNO_2_. The crystalline samples were heated from room temperature to 200 °C and diffractograms taken at 5 °C intervals. For both samples, amorphization was observed. After amorphization, no further changes were observed until melting of the sample. Figure 8 and Figure 9 report the two-dimensional plots obtained for Moxi·2*i*PrOH and Moxi·1.5MeNO_2_, respectively. In the literature, the slow desolvation with concomitant recrystallization to form I has been reported for the EtOH solvate [18] in water/ethanol mixture, concluding that water plays a crucial role in this transformation. We did not observe the appearance of form I while performing the VT-PXRD experiment with Moxi·2*i*PrOH and Moxi·1.5MeNO_2_, drawing the same conclusion.

Moreover, slow recrystallization of amorphous moxidectin at high temperature has also been reported in the literature [13]. To verify this, we performed an additional experiment heating the amorphous moxidectin for a prolonged time (5 h) between 170 and 190 °C (Figure S1). However, no formation of a new crystalline phase could be detected in these conditions.

## 3. Experimental Section

### 3.1. Materials and Methods—Crystallization

Moxidectin was purchased from Merck-Sigma Aldrich. The solvents were purchased from Carlo Erba Reagent and used without further purification.

The search for experimental conditions for the crystallization of moxidectin was undertaken by systematic variation of solvents and temperature. Protic and aprotic polar solvents such as water, methanol, 1-propanol, 2-propanol, 1-butanol, ethanol, nitromethane (MeNO_2_), acetone, acetonitrile, THF, as well as nonpolar solvents such as toluene, cyclohexane, diisopropylether, MTBE and dichloromethane were tested.

Moxidectin was suspended in the respective solvent at different concentrations and the mixture was gently heated until complete dissolution. The solutions were placed in a closed vial to avoid evaporation of the solvent and stored at 4 °C or 25 °C. This operation was carried out in the dark to avoid photodegradation of moxidectin [24]. Crystals were obtained from the ethanol, 2-propanol and nitromethane solutions stored at 4 °C. No crystals were obtained from solutions stored at 25 °C except for the ones in 2-propanol.

### 3.2. Single Crystal X-ray Diffraction

Single crystal X-ray diffraction (SC-XRD) data were collected with an AXS APEXII CCD area-detector diffractometer (BRUKER). The radiation source was a molybdenum anode (Mo-Kα, λ = 0.71073 Å) with the generator working at 50 kV and 30 mA. The data reduction was carried out with CrysAlis Pro [25] version 1.171.40.53 using an empirical absorption correction with spherical harmonics (SCALE3 ABSPACK). The structure was solved by dual space methods with SHELXT-2017 [26] and refined with SHELXL-2018 [27] using the WinGX program suite [28]. Structure refinement was done using full-matrix least-square routines against F^2^. All hydrogen atoms with the exception of Hydroxyl-H atoms were calculated on idealized positions. The pictures were generated with the programs ORTEP-3 [28] (representation with anisotropic displacement parameters), MERCURY [29] (comparison of molecular conformations) and DIAMOND [30] (all other images). Hydrogen atoms besides for OH groups were omitted for clarity in these representations. CCDC 2089844 (Moxi·2EtOH), CCDC 2089842 (Moxi·2*i*PrOH), CCDC 2089843 (Moxi·2*i*PrOH, room temperature), CCDC 2089841 (Moxi·1.5MeNO_2_) contain the supplementary crystallographic data in the Supplementary Materials for this paper. These data and additional information can be obtained free of charge via https://summary.ccdc.cam.ac.uk/structure-summary-form (or from the Cambridge Crystallographic Data Centre, 12 Union Road, Cambridge CB2 1EZ, UK; Fax: (+44)1223-336-033; or deposit@ccdc.cam.uk). Table 2 contains the summary of the crystallographic data for each structure. The calculation of the theoretical powder pattern from the single crystal structures was performed using the powder diffraction simulation tool implemented in Mercury [29].

### 3.3. Variable-Temperature X-ray Powder Diffraction (VT-XRPD) Analysis

Diffraction experiments were performed using Cu-Kα radiation (λ = 1.5418 Å) on a vertical-scan Bruker AXS D8 Advance diffractometer in θ:θ mode, equipped with a Goebel Mirror and a Bruker Lynxeye linear Position Sensitive Detector (PSD), with the following optics: primary and secondary Soller slits, 2.3° and 2.5°, respectively; divergence slit, 0.1°; receiving slit, 2.82°. Generator setting: 40 kV, 40 mA. The nominal resolution for the present set-up is 0.08° 2θ (FWHM of the α1 component) for the LaB_6_ peak at about 21.3° (2θ). VT-XRPD experiments were carried out by coupling a custom-made sample heater, assembled by Officina Elettrotecnica di Tenno, Ponte Arche, Italy, to the instrumental set-up described above. For that, a powdered microcrystalline sample was ground in an agate mortar and was deposited in the hollow of a quartz zero-background plate framed by an aluminum skeleton. The data were acquired within a sensible, low-angle 2θ range. The samples were heated in situ in the temperature range 20–130 °C (steps of 10 °C, Moxi·1.5MeNO_2_), 20–110 °C (steps of 5 °C, Moxi·2*i*PrOH) and 170–200 °C (over the course of 5 h, amorphous Moxi). Before each measurement we waited 5 minutes for temperature equilibration, while each measurement took about 8 minutes to be completed (0.02°/step and 0.5 s/step, as experimental conditions for the data collection). The VT diffractograms are depicted in Figure 8 and Figure 9 and Figure S1. The diffraction patterns were elaborated with the software EVA [31]. Performing the experiments both in air and in nitrogen atmosphere gave identical results.

### 3.4. Quantum Chemical Calculations

The calculations were carried with DFT using the software suite CRYSTAL-17 [32], applying the B3LYP functional [33,34] in combination with the basis set 6–31 G [35,36,37]. Furthermore, the atom-pairwise dispersion correction D3 [38] was used and the convergence criterion set to 10^−8^ Hartree.

The input for the optimization of the hypothetical solvatomorph of 1-butanol was created by modifying the crystal structure of the ethanol solvatomorph through replacing hydrogen atoms in the solvents by ethyl groups. The optimized crystal structure is isostructural to the solvatomorphs of ethanol and 2-propanol. The obtained unit cell parameters were a = 11.4065, b = 8.9996, c = 21.5342, β = 93.7797°; the theoretical temperature corresponds to 0 K. Further details, including the structure model, are listed in the Supplementary Material.

## 4. Conclusions

In this paper we clarified the solid-state chemistry of moxidectin API. Through SC-XRD analysis we were able to report a crystallographic characterization of moxidectin solvatomorphs containing two equivalents of ethanol and 2-propanol. Furthermore, a new solvatomorph with nitromethane was obtained and structurally characterized. Our structural analysis confirmed the isostructural nature of the alcoholate solvatomorphs (Moxi·2ROH, R = Et, *i*Pr). Moreover, a deep analysis on their crystal structures and literature reports, together with the aid of quantum chemical calculations, lead to the conclusion that the claimed solvatomorph with 1-butanol likely possesses 2 instead of 1.5 solvent molecules in its crystal structure, being isostructural to the other alcohol-based solvatomorphs. Careful analysis of the intermolecular interactions in the reported crystal structures revealed a substantial relationship between the molecular conformation of moxidectin and the hydrogen bonds present in the solid-state of its solvatomorphs. This work offers a comprehensive view of the solvatomorphism of moxidectin and provides important information for the manufacture of medicinal products of this active pharmaceutical ingredient, which are useful to meet the strict requisites of efficacy, safety, quality mandatory for the marketing authorization.

## Figures and Tables

**Table 1:** Synoptic collection of moxidectin crystal forms as well as structural data and literature reports.

Crystal Form	First Report	Structure Report
Form I (Moxi, unsolvated)	Ref. [12]	Ref. [12], CSD: GETBOW
Amorphous (Moxi, unsolvated)	Ref. [13]	–
Moxi·2EtOH	Ref. [13]	this work
Mox·2*i*PrOH	Ref. [13]	this work
Moxi·2*n*BuOH	Ref. [13] (reported as Moxi·1.5*n*BuOH)	–
Moxi·1.5MeNO_2_	this work	this work

**Table 2:** Summary of crystallographic data.

Compound	Moxi·2EtOH	Moxi·2*i*PrOH	Moxi·1.5MeNO_2_
**Formula**	C_40_H_65_NO_10_	C_43_H_69_NO_10_	C_38.23_H_56.70_N_2.23_O_10.47_
**FW**	731.94	759.99	715.05
**Crystal System**	Monoclinic	Monoclinic	Orthorhombic
**Space group**	*P*2_1_	*P*2_1_	*P*2_1_2_1_2
***a* (Å)**	11.1913(4)	11.4417(3)	13.9453(5)
***b* (Å)**	8.8440(3)	8.9507(2)	19.8096(6)
***c* (Å)**	21.9594(8)	21.7210(6)	15.2680(6)
***α* (°)**	90	90	90
***β* (°)**	93.747(3)	92.301(3)	90
***γ* (°)**	90	90	90
***V* (Å)^3^**	2168.80(13)	2222.68(10)	4217.8(3)
***Z***	2	2	4
**Temperature (K)**	120(2)	120(2)	100(2)
**ρ_(calcd)_ (g·cm^–3^)**	1.121	1.136	1.126
**μ (mm^–1^)**	0.079	0.079	0.081
**F(000)**	796	828	1542
**Crystal size (mm^3^)**	0.150 × 0.120 × 0.100	0.200 × 0.100 × 0.100	0.120 × 0.120 × 0.030
***θ* range (°)**	1.824 to 31.571	1.781 to 31.719	1.684 to 26.372
**Limiting indices**	−16 ≤ h ≤ 16, −12 ≤ k ≤ 13, −32 ≤ l ≤ 32	−16 ≤ h ≤ 16, −13 ≤ k ≤ 13, −31 ≤ l ≤ 30	−17 ≤ h ≤ 17, −24 ≤ k ≤ 24, −19 ≤ l ≤ 19
**Reflns collected/unique ^a^**	25503/13011 [R_int_ = 0.0249]	26404/13370 [R_int_ = 0.0180]	36172/8640 [R_int_ = 0.0244]
**Data/restraints/param**	13011/9/485	13370/9/538	8638/2/508
**Completeness to** ***θ* = 25.242° (%)**	100.0	100.0	100.00
**Max. and min. transmission**	1.00000 and 0.96333	1.00000 and 0.90598	1.00000 and 0.61508
**Final R indices** **[I > 2σ(I)] ^b^**	R_1_ = 0.0525, wR_2_ = 0.1160	R_1_ = 0.0448, wR_2_ = 0.1112	R_1_ = 0.0536, wR_2_ = 0.1326
**R indices (all data)**	R_1_ = 0.0720, wR_2_ = 0.1254	R_1_ = 0.0549, wR_2_ = 0.1169	R_1_ = 0.0584, wR_2_ = 0.1357
**GooF ^c^ on F^2^**	1.013	1.021	1.100
**Largest diff peak** **and hole (e·Å** **^−3^)**	0.652 and −0.426	0.355 and −0.344	0.601 and −0.435

^a^ R_int_ = Σ|F_o_^2^ − F_o_^2^(mean)|/ΣF_o_^2^, ^b^ R_1_ = Σ||F_o_| − |F_c_||/Σ|F_o_|, wR_2_ = {Σ[*w*(F_o_^2^ − F_c_^2^)^2^]/Σ[*w*(F_o_^2^)^2^]}^1/2^, ^c^ GooF = {S/(*n −p*)}^1/2^ = {Σ[*w*(F_o_^2^ − F_c_^2^)^2^]/(*n − p*)}^1/2^.

## Data Availability

Data are available from the authors upon reasonable request.

## Supplementary Materials

The following are available online. Variable-temperature X-ray powder diffraction (VT-XRPD) of amorphous moxidectin (Figure S1).; distances [Å] and type for strong and weak hydrogen bonds observed in form I and in the solvatomorphs discussed in this work (Table S1) and additional information on quantum chemical calculations.

## Sample Availability

Samples are not available from the authors.
