# Supplementary material for: Solvatomorphism of Moxidectin
Source: Molecules. 2021 Aug 11;26(16):4869. doi: 10.3390/molecules26164869 (PMC8398095; doi:10.3390/molecules26164869)

checkCIF/PLATON report

Structure factors have been supplied for datablock(s) vale30

THIS REPORT IS FOR GUIDANCE ONLY. IF USED AS PART OF A REVIEW PROCEDURE FOR PUBLICATION, IT SHOULD NOT REPLACE THE EXPERTISE OF AN EXPERIENCED CRYSTALLOGRAPHIC REFEREE.

No syntax errors found.   CIF dictionary   Interpreting this report

Datablock: vale30

```
Bond precision:   C-C = 0.0049 A            Wavelength=0.71073

Cell:             a=13.9453(5)      b=19.8096(6)      c=15.2680(6)
                  alpha=90          beta=90           gamma=90
Temperature:      100 K
```

	Calculated	Reported
Volume	4217.8(3)	4217.8(3)
Space group	P 21 21 2	P 21 21 2
Hall group	P 2 2ab	P 2 2ab
Moiety formula	C37 H53 N O8, 1.233(C H3 N O2)	C37 H53 N O8, 1.23(C H3 N O2)
Sum formula	C38.23 H56.70 N2.23 O10.47	C38.23 H56.70 N2.23 O10.47
Mr	715.07	715.05
Dx,g cm-3	1.126	1.126
Z	4	4
Mu (mm-1)	0.081	0.081
F000	1541.8	1542.0
F000’	1542.62	
h,k,lmax	17,24,19	17,24,19
Nref	8638[4794]	8640
Tmin,Tmax	0.990,0.998	0.615,1.000
Tmin’	0.990	

```
Correction method= # Reported T Limits: Tmin=0.615 Tmax=1.000
AbsCorr = MULTI-SCAN

Data completeness= 1.80/1.00      Theta(max)= 26.372

R(reflections)= 0.0536( 7995)     wR2(reflections)= 0.1357( 8640)

S = 1.100                  Npar= 539
```

The following ALERTS were generated. Each ALERT has the format
test-name_ALERT_alert-type_alert-level.
Click on the hyperlinks for more details of the test.

Alert level C

DIFMX02_ALERT_1_C The maximum difference density is > 0.1*ZMAX*0.75
The relevant atom site should be identified.

Author Response: The highest residual electron density peak is located close to O1 and a split position refinement seems reasonable. However, the bond pa- rameters obtained for the second site are not reasonable. When re- fined, the C-O a bond length for the second O1 site is 1.427. This can be adjusted using SADI. However, the C2-C1-O1-O6 torsion angle for the second O1 site is 136 which is in stark contrast to the ex- pected 180 for a carbonyl group. Using restraints to model this did not improve this situation and so we decided to not model this split. Presumably, the Q peak is caused by an artefact instead of a second atomic position.

PLAT213_ALERT_2_C Atom O1 has ADP max/min Ratio ..... 3.8 prolat

Author Response: The atoms O1, C29 and C30 have a somewhat large ADP max/min ratio. However, they were still refined anisotropically as a split disorder could not be reasonably refined

PLAT213_ALERT_2_C Atom C29 has ADP max/min Ratio ..... 3.1 prolat

Author Response: The atoms O1, C29 and C30 have a somewhat large ADP max/min ratio. However, they were still refined anisotropically as a split disorder could not be reasonably refined

```
PLAT220_ALERT_2_C NonSolvent   Resd 1  C   Ueq(max)/Ueq(min) Range        5.0 Ratio
PLAT220_ALERT_2_C NonSolvent   Resd 1  O   Ueq(max)/Ueq(min) Range        4.2 Ratio
PLAT222_ALERT_3_C NonSolvent Resd 1  H   Uiso(max)/Uiso(min) Range        5.7 Ratio
PLAT242_ALERT_2_C Low    'MainMol' Ueq as Compared to Neighbors of         C1 Check
PLAT242_ALERT_2_C Low    'MainMol' Ueq as Compared to Neighbors of        C28 Check
PLAT340_ALERT_3_C Low Bond Precision on  C-C Bonds ...............    0.00489 Ang.
PLAT601_ALERT_2_C Unit Cell Contains Solvent Accessible VOIDS of .         79 Ang**3
```

Author Response: The crystal structure contains small solvent accessible voids. Howev- er, they most likely results from a loose packing in the crystal structure and do not contain any additional solvent molecules. In ac- cordance with that, when applying the SQUEEZE routine implemented in PLATON to the current model, an insignificant electron count of only 10 electrons for the entire unit cell is obtained.

```
PLAT906_ALERT_3_C Large K Value in the Analysis of Variance ......      2.066 Check
PLAT918_ALERT_3_C Reflection(s) with I(obs) much Smaller I(calc) .          4 Check
```

Alert level G

```
FORMU01_ALERT_1_G  There is a discrepancy between the atom counts in the
            _chemical_formula_sum and _chemical_formula_moiety. This is
            usually due to the moiety formula being in the wrong format.
            Atom count from _chemical_formula_sum:   C38.23 H56.7 N2.23 O10.47
            Atom count from _chemical_formula_moiety:C38.23 H56.69 N2.23 O10.46
PLAT002_ALERT_2_G Number of Distance or Angle Restraints on AtSite          4 Note
PLAT042_ALERT_1_G Calc. and Reported Moiety Formula Strings Differ     Please Check
```

Author Response: Caused by the disorder of the three loosely packed nitromethane sol- vent molecules about a twofold axis. The molecules could unambiguous- ly be identified. Their disorder caused several problems with the au- tomatic calculation of the sum and moiety formula as well the Z val- ue.

```
PLAT068_ALERT_1_G Reported F000 Differs from Calcd (or Missing)...     Please Check
PLAT172_ALERT_4_G The CIF-Embedded .res File Contains DFIX Records          1 Report
PLAT300_ALERT_4_G Atom Site Occupancy of O9         Constrained at        0.5 Check
```

Author Response: Caused by the disorder of the three loosely packed nitromethane sol- vent molecules about a twofold axis. Unusually large ADPs in combina- tion with large regions of negative electron density and a squeezed electron count of a model without solvent molecules (_refine_special_details) suggested partial occupancy. The occupancy of one nitromethane molecule (N2,O9,O10) was refined to 99% and was thus fixed at full occupancy.

```
PLAT300_ALERT_4_G Atom Site Occupancy of O10        Constrained at        0.5 Check
```

Author Response: Caused by the disorder of the three loosely packed nitromethane sol- vent molecules about a twofold axis. Unusually large ADPs in combina- tion with large regions of negative electron density and a squeezed electron count of a model without solvent molecules (_refine_special_details) suggested partial occupancy. The occupancy of one nitromethane molecule (N2,O9,O10) was refined to 99% and was thus fixed at full occupancy.

```
PLAT300_ALERT_4_G Atom Site Occupancy of N2         Constrained at        0.5 Check
```

Author Response: Caused by the disorder of the three loosely packed nitromethane sol- vent molecules about a twofold axis. Unusually large ADPs in combina- tion with large regions of negative electron density and a squeezed electron count of a model without solvent molecules (_refine_special_details) suggested partial occupancy. The occupancy of one nitromethane molecule (N2,O9,O10) was refined to 99% and was thus fixed at full occupancy.

```
PLAT300_ALERT_4_G Atom Site Occupancy of C38        Constrained at        0.5 Check
```

Author Response: Caused by the disorder of the three loosely packed nitromethane sol- vent molecules about a twofold axis. Unusually large ADPs in combina- tion with large regions of negative electron density and a squeezed electron count of a model without solvent molecules (_refine_special_details) suggested partial occupancy. The occupancy of one nitromethane molecule (N2,O9,O10) was refined to 99% and was thus fixed at full occupancy.

```
PLAT300_ALERT_4_G Atom Site Occupancy of H54        Constrained at        0.5 Check
```

Author Response: Caused by the disorder of the three loosely packed nitromethane sol- vent molecules about a twofold axis. Unusually large ADPs in combina- tion with large regions of negative electron density and a squeezed electron count of a model without solvent molecules (_refine_special_details) suggested partial occupancy. The occupancy of one nitromethane molecule (N2,O9,O10) was refined to 99% and was thus fixed at full occupancy.

```
PLAT300_ALERT_4_G Atom Site Occupancy of H55        Constrained at        0.5 Check
```

Author Response: Caused by the disorder of the three loosely packed nitromethane sol- vent molecules about a twofold axis. Unusually large ADPs in combina- tion with large regions of negative electron density and a squeezed electron count of a model without solvent molecules (_refine_special_details) suggested partial occupancy. The occupancy of one nitromethane molecule (N2,O9,O10) was refined to 99% and was thus fixed at full occupancy.

```
PLAT300_ALERT_4_G Atom Site Occupancy of H56        Constrained at        0.5 Check
```

Author Response: Caused by the disorder of the three loosely packed nitromethane sol- vent molecules about a twofold axis. Unusually large ADPs in combina- tion with large regions of negative electron density and a squeezed electron count of a model without solvent molecules (_refine_special_details) suggested partial occupancy. The occupancy of one nitromethane molecule (N2,O9,O10) was refined to 99% and was thus fixed at full occupancy.

```
PLAT302_ALERT_4_G Anion/Solvent/Minor-Residue Disorder (Resd  2  )       100% Note
```

Author Response: Caused by the disorder of the three loosely packed nitromethane sol- vent molecules about a twofold axis. Unusually large ADPs in combina- tion with large regions of negative electron density and a squeezed electron count of a model without solvent molecules (_refine_special_details) suggested partial occupancy. The occupancy of two of the three nitromethane molecule (N3,O11,O12,N4,O13,O14) was refined to values significantly lower that 100%.

```
PLAT302_ALERT_4_G Anion/Solvent/Minor-Residue Disorder (Resd  3  )       100% Note
```

Author Response: Caused by the disorder of the three loosely packed nitromethane sol- vent molecules about a twofold axis. Unusually large ADPs in combina- tion with large regions of negative electron density and a squeezed electron count of a model without solvent molecules (_refine_special_details) suggested partial occupancy. The occupancy of two of the three nitromethane molecule (N3,O11,O12,N4,O13,O14) was refined to values significantly lower that 100%.

```
PLAT302_ALERT_4_G Anion/Solvent/Minor-Residue Disorder (Resd  4  )       100% Note
```

Author Response: Caused by the disorder of the three loosely packed nitromethane sol- vent molecules about a twofold axis. Unusually large ADPs in combina- tion with large regions of negative electron density and a squeezed electron count of a model without solvent molecules (_refine_special_details) suggested partial occupancy. The occupancy of two of the three nitromethane molecule (N3,O11,O12,N4,O13,O14) was refined to values significantly lower that 100%.

```
PLAT304_ALERT_4_G Non-Integer Number of Atoms in ..... (Resd  2  )       3.50 Check
```

Author Response: Caused by the disorder of the three loosely packed nitromethane sol- vent molecules about a twofold axis. Unusually large ADPs in combina- tion with large regions of negative electron density suggested par- tial occupancy. The occupancy of two of the three nitromethane mole- cule (N3,O11,O12,N4,O13,O14) was refined to values significantly low- er that 100%.

```
PLAT304_ALERT_4_G Non-Integer Number of Atoms in ..... (Resd  3  )       3.07 Check
```

Author Response: Caused by the disorder of the three loosely packed nitromethane sol- vent molecules about a twofold axis. Unusually large ADPs in combina- tion with large regions of negative electron density suggested par- tial occupancy. The occupancy of two of the three nitromethane mole- cule (N3,O11,O12,N4,O13,O14) was refined to values significantly low- er that 100%.

```
PLAT304_ALERT_4_G Non-Integer Number of Atoms in ..... (Resd  4  )       2.06 Check
```

Author Response: Caused by the disorder of the three loosely packed nitromethane sol- vent molecules about a twofold axis. Unusually large ADPs in combina- tion with large regions of negative electron density suggested par- tial occupancy. The occupancy of two of the three nitromethane mole- cule (N3,O11,O12,N4,O13,O14) was refined to values significantly low- er that 100%.

```
PLAT380_ALERT_4_G Incorrectly? Oriented X(sp2)-Methyl Moiety .....        C38 Check
PLAT395_ALERT_2_G Deviating  X-O-Y    Angle From 120 for O8             108.7 Degree
PLAT398_ALERT_2_G Deviating  C-O-C    Angle From 120 for O3             105.5 Degree
PLAT789_ALERT_4_G Atoms with Negative _atom_site_disorder_group  #         21 Check
```

Author Response: Caused by the disorder of the three loosely packed nitromethane sol- vent molecules about a twofold axis of which two are only partially occupied.

```
PLAT791_ALERT_4_G Model has Chirality at C2          (Sohnke SpGr)          R Verify
```

Author Response: The absolute structure of the molecule is known and was not deter- mined by anomalous-dispersion effects. The sample was a weak anomalous scatterer. The absolute structure parameter was calculated to be -0.5(2) which is meaningless for the determination of the absolute structure. It was thus deleted from the *.cif file.

```
PLAT791_ALERT_4_G Model has Chirality at C5          (Sohnke SpGr)          R Verify
```

Author Response: The absolute structure of the molecule is known and was not deter- mined by anomalous-dispersion effects. The sample was a weak anomalous scatterer. The absolute structure parameter was calculated to be -0.5(2) which is meaningless for the determination of the absolute structure. It was thus deleted from the *.cif file.

```
PLAT791_ALERT_4_G Model has Chirality at C6          (Sohnke SpGr)          R Verify
```

Author Response: The absolute structure of the molecule is known and was not deter- mined by anomalous-dispersion effects. The sample was a weak anomalous scatterer. The absolute structure parameter was calculated to be -0.5(2) which is meaningless for the determination of the absolute structure. It was thus deleted from the *.cif file.

```
PLAT791_ALERT_4_G Model has Chirality at C7          (Sohnke SpGr)          S Verify
```

Author Response: The absolute structure of the molecule is known and was not deter- mined by anomalous-dispersion effects. The sample was a weak anomalous scatterer. The absolute structure parameter was calculated to be -0.5(2) which is meaningless for the determination of the absolute structure. It was thus deleted from the *.cif file.

```
PLAT791_ALERT_4_G Model has Chirality at C12         (Sohnke SpGr)          R Verify
```

Author Response: The absolute structure of the molecule is known and was not deter- mined by anomalous-dispersion effects. The sample was a weak anomalous scatterer. The absolute structure parameter was calculated to be -0.5(2) which is meaningless for the determination of the absolute structure. It was thus deleted from the *.cif file.

```
PLAT791_ALERT_4_G Model has Chirality at C17         (Sohnke SpGr)          R Verify
```

Author Response: The absolute structure of the molecule is known and was not deter- mined by anomalous-dispersion effects. The sample was a weak anomalous scatterer. The absolute structure parameter was calculated to be -0.5(2) which is meaningless for the determination of the absolute structure. It was thus deleted from the *.cif file.

```
PLAT791_ALERT_4_G Model has Chirality at C19         (Sohnke SpGr)          S Verify
```

Author Response: The absolute structure of the molecule is known and was not deter- mined by anomalous-dispersion effects. The sample was a weak anomalous scatterer. The absolute structure parameter was calculated to be -0.5(2) which is meaningless for the determination of the absolute structure. It was thus deleted from the *.cif file.

```
PLAT791_ALERT_4_G Model has Chirality at C21         (Sohnke SpGr)          R Verify
```

Author Response: The absolute structure of the molecule is known and was not deter- mined by anomalous-dispersion effects. The sample was a weak anomalous scatterer. The absolute structure parameter was calculated to be -0.5(2) which is meaningless for the determination of the absolute structure. It was thus deleted from the *.cif file.

```
PLAT791_ALERT_4_G Model has Chirality at C24         (Sohnke SpGr)          S Verify
```

Author Response: The absolute structure of the molecule is known and was not deter- mined by anomalous-dispersion effects. The sample was a weak anomalous scatterer. The absolute structure parameter was calculated to be -0.5(2) which is meaningless for the determination of the absolute structure. It was thus deleted from the *.cif file.

```
PLAT791_ALERT_4_G Model has Chirality at C25         (Sohnke SpGr)          S Verify
```

Author Response: The absolute structure of the molecule is known and was not deter- mined by anomalous-dispersion effects. The sample was a weak anomalous scatterer. The absolute structure parameter was calculated to be -0.5(2) which is meaningless for the determination of the absolute structure. It was thus deleted from the *.cif file.

```
PLAT860_ALERT_3_G Number of Least-Squares Restraints .............          2 Note
PLAT910_ALERT_3_G Missing # of FCF Reflection(s) Below Theta(Min).          1 Note
PLAT965_ALERT_2_G The SHELXL WEIGHT Optimisation has not Converged     Please Check
PLAT978_ALERT_2_G Number C-C Bonds with Positive Residual Density.          5 Info
```

```
  0 ALERT level A = Most likely a serious problem - resolve or explain
  0 ALERT level B = A potentially serious problem, consider carefully
 12 ALERT level C = Check. Ensure it is not caused by an omission or oversight
 36 ALERT level G = General information/check it is not something unexpected

  4 ALERT type 1 CIF construction/syntax error, inconsistent or missing data
 12 ALERT type 2 Indicator that the structure model may be wrong or deficient
  6 ALERT type 3 Indicator that the structure quality may be low
 26 ALERT type 4 Improvement, methodology, query or suggestion
  0 ALERT type 5 Informative message, check
```

It is advisable to attempt to resolve as many as possible of the alerts in all categories. Often the minor alerts point to easily fixed oversights, errors and omissions in your CIF or refinement strategy, so attention to these fine details can be worthwhile. In order to resolve some of the more serious problems it may be necessary to carry out additional measurements or structure refinements. However, the purpose of your study may justify the reported deviations and the more serious of these should normally be commented upon in the discussion or experimental section of a paper or in the "special_details" fields of the CIF. checkCIF was carefully designed to identify outliers and unusual parameters, but every test has its limitations and alerts that are not important in a particular case may appear. Conversely, the absence of alerts does not guarantee there are no aspects of the results needing attention. It is up to the individual to critically assess their own results and, if necessary, seek expert advice.

Publication of your CIF in IUCr journals

A basic structural check has been run on your CIF. These basic checks will be run on all CIFs submitted for publication in IUCr journals (*Acta Crystallographica*, *Journal of Applied Crystallography*, *Journal of Synchrotron Radiation*); however, if you intend to submit to *Acta Crystallographica Section C* or *E* or *IUCrData*, you should make sure that full publication checks are run on the final version of your CIF prior to submission.

Publication of your CIF in other journals

Please refer to the *Notes for Authors* of the relevant journal for any special instructions relating to CIF submission.

PLATON version of 13/07/2021; check.def file version of 13/07/2021

Datablock vale30 - ellipsoid plot

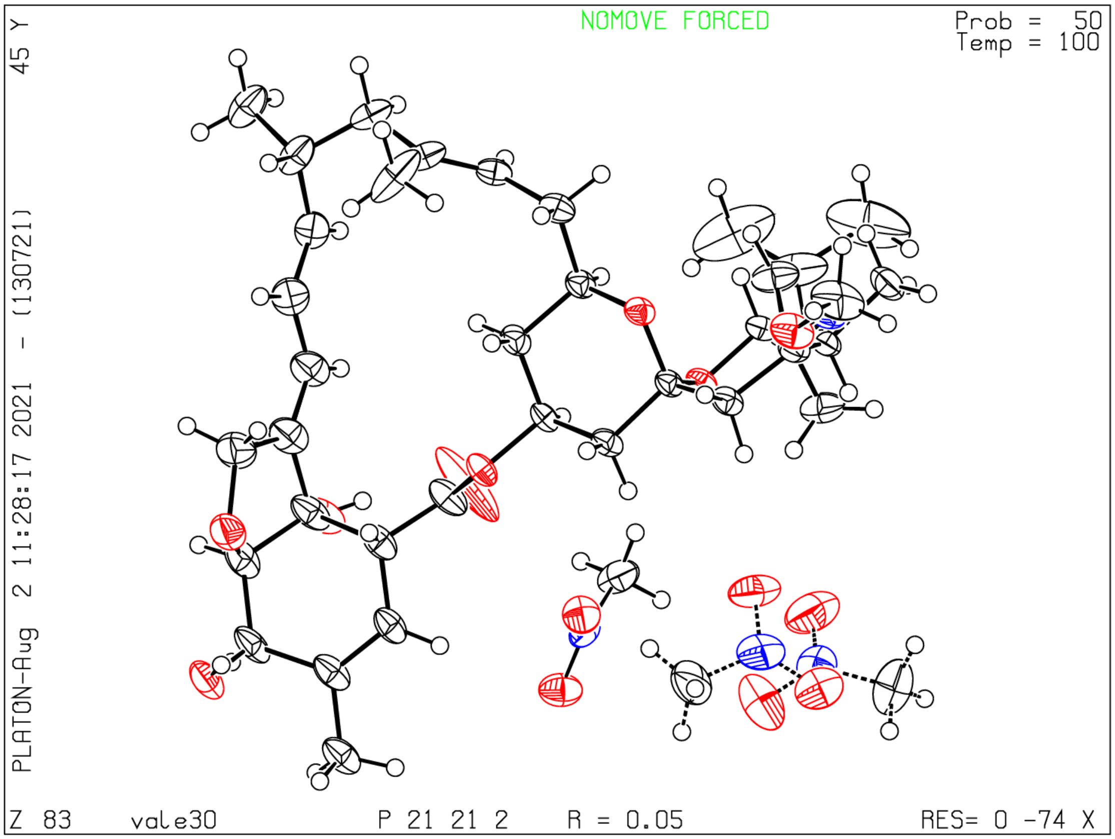

Supplement: Supplementary file 1 [file molecules-26-04869-s001.zip › molecules-1312668-supplementary/ESI/Moxi.1.5MeNO2_checkcif.pdf]
