# Supplementary material for: Solvatomorphism of Moxidectin
Source: Molecules. 2021 Aug 11;26(16):4869. doi: 10.3390/molecules26164869 (PMC8398095; doi:10.3390/molecules26164869)

checkCIF/PLATON report

Structure factors have been supplied for datablock(s) vale25

THIS REPORT IS FOR GUIDANCE ONLY. IF USED AS PART OF A REVIEW PROCEDURE FOR PUBLICATION, IT SHOULD NOT REPLACE THE EXPERTISE OF AN EXPERIENCED CRYSTALLOGRAPHIC REFEREE.

No syntax errors found. CIF dictionary Interpreting this report

Datablock: vale25

```
Bond precision:  C-C = 0.0035 A          Wavelength=0.71073

Cell:            a=11.5186(5)     b=9.1524(4)       c=21.8079(9)
                 alpha=90         beta=93.005(4)    gamma=90
Temperature:     293 K

                Calculated                  Reported
Volume          2295.89(17)                 2295.89(17)
Space group     P 21                        P 21
Hall group      P 2yb                       P 2yb
Moiety formula  C37 H53 N O8 [+ solvent]    C37 H53 N1 O8
Sum formula     C37 H53 N O8 [+ solvent]    C37 H53 N O8
Mr              639.80                      639.80
Dx,g cm-3       0.925                       0.925
Z               2                           2
Mu (mm-1)       0.064                       0.064
F000            692.0                       692.0
F000'           692.34
h,k,lmax        15,12,29                    15,12,29
Nref            11400[ 6051]                11396
Tmin,Tmax       0.992,0.994                 0.887,1.000
Tmin'           0.990

Correction method= # Reported T Limits: Tmin=0.887 Tmax=1.000
AbsCorr = MULTI-SCAN

Data completeness= 1.88/1.00      Theta(max)= 28.282

R(reflections)= 0.0413( 8779)     wR2(reflections)= 0.1133( 11396)

S = 1.052                  Npar= 426
```

```
The following ALERTS were generated. Each ALERT has the format
       test-name_ALERT_alert-type_alert-level.
Click on the hyperlinks for more details of the test.
```

Alert level B

```
PLAT420_ALERT_2_B D-H Bond Without Acceptor  O2       --H52      .     Please Check
```

Author Response: Caused by applying SQUEEZE. There is a hydrogen bond the solvent mol- ecule. For further details see the low-temperature measurement or the article.

Alert level C

```
PLAT242_ALERT_2_C Low    'MainMol' Ueq as Compared to Neighbors of        C14 Check
PLAT242_ALERT_2_C Low    'MainMol' Ueq as Compared to Neighbors of        C28 Check
PLAT934_ALERT_3_C Number of (Iobs-Icalc)/Sigma(W) > 10 Outliers ..          1 Check
```

Alert level G

```
PLAT007_ALERT_5_G Number of Unrefined Donor-H Atoms .............           2 Report
PLAT199_ALERT_1_G Reported _cell_measurement_temperature ..... (K)        293 Check
PLAT200_ALERT_1_G Reported   _diffrn_ambient_temperature ..... (K)        293 Check
PLAT395_ALERT_2_G Deviating  X-O-Y    Angle From 120 for O8             108.4 Degree
PLAT398_ALERT_2_G Deviating  C-O-C    Angle From 120 for O3             106.5 Degree
PLAT606_ALERT_4_G Solvent Accessible VOID(S) in Structure ........          ! Info
```

Author Response: SQUEEZE was applied. See response above.

```
PLAT791_ALERT_4_G Model has Chirality at C2          (Sohnke SpGr)          R Verify
```

Author Response: The absolute structure of the molecule is known and was not deter- mined by anomalous-dispersion effects. The sample was a weak anoma- lous scatterer. The absolute structure parameter was calculated to be 0.2(3) which is meaningless for the determination of the absolute structure. It was thus deleted from the *.cif file.

```
PLAT791_ALERT_4_G Model has Chirality at C5          (Sohnke SpGr)          R Verify
```

Author Response: The absolute structure of the molecule is known and was not deter- mined by anomalous-dispersion effects. The sample was a weak anoma- lous scatterer. The absolute structure parameter was calculated to be 0.2(3) which is meaningless for the determination of the absolute structure. It was thus deleted from the *.cif file.

```
PLAT791_ALERT_4_G Model has Chirality at C6          (Sohnke SpGr)          R Verify
```

Author Response: The absolute structure of the molecule is known and was not deter- mined by anomalous-dispersion effects. The sample was a weak anoma- lous scatterer. The absolute structure parameter was calculated to be 0.2(3) which is meaningless for the determination of the absolute structure. It was thus deleted from the *.cif file.

```
PLAT791_ALERT_4_G Model has Chirality at C7          (Sohnke SpGr)          S Verify
```

Author Response: The absolute structure of the molecule is known and was not deter- mined by anomalous-dispersion effects. The sample was a weak anoma- lous scatterer. The absolute structure parameter was calculated to be 0.2(3) which is meaningless for the determination of the absolute structure. It was thus deleted from the *.cif file.

```
PLAT791_ALERT_4_G Model has Chirality at C12         (Sohnke SpGr)          R Verify
```

Author Response: The absolute structure of the molecule is known and was not deter- mined by anomalous-dispersion effects. The sample was a weak anoma- lous scatterer. The absolute structure parameter was calculated to be 0.2(3) which is meaningless for the determination of the absolute structure. It was thus deleted from the *.cif file.

```
PLAT791_ALERT_4_G Model has Chirality at C17         (Sohnke SpGr)          R Verify
```

Author Response: The absolute structure of the molecule is known and was not deter- mined by anomalous-dispersion effects. The sample was a weak anoma- lous scatterer. The absolute structure parameter was calculated to be 0.2(3) which is meaningless for the determination of the absolute structure. It was thus deleted from the *.cif file.

```
PLAT791_ALERT_4_G Model has Chirality at C19         (Sohnke SpGr)          S Verify
```

Author Response: The absolute structure of the molecule is known and was not deter- mined by anomalous-dispersion effects. The sample was a weak anoma- lous scatterer. The absolute structure parameter was calculated to be 0.2(3) which is meaningless for the determination of the absolute structure. It was thus deleted from the *.cif file.

```
PLAT791_ALERT_4_G Model has Chirality at C21         (Sohnke SpGr)          R Verify
```

Author Response: The absolute structure of the molecule is known and was not deter- mined by anomalous-dispersion effects. The sample was a weak anoma- lous scatterer. The absolute structure parameter was calculated to be 0.2(3) which is meaningless for the determination of the absolute structure. It was thus deleted from the *.cif file.

```
PLAT791_ALERT_4_G Model has Chirality at C24         (Sohnke SpGr)          S Verify
```

Author Response: The absolute structure of the molecule is known and was not deter- mined by anomalous-dispersion effects. The sample was a weak anoma- lous scatterer. The absolute structure parameter was calculated to be 0.2(3) which is meaningless for the determination of the absolute structure. It was thus deleted from the *.cif file.

```
PLAT791_ALERT_4_G Model has Chirality at C25         (Sohnke SpGr)          S Verify
```

Author Response: The absolute structure of the molecule is known and was not deter- mined by anomalous-dispersion effects. The sample was a weak anoma- lous scatterer. The absolute structure parameter was calculated to be 0.2(3) which is meaningless for the determination of the absolute structure. It was thus deleted from the *.cif file.

```
PLAT869_ALERT_4_G ALERTS Related to the Use of SQUEEZE Suppressed           ! Info
```

Author Response: The same crystal structure has been determined at lower temperature where the atomic sites could be determined reliably. This crystal structure was measured at room temperature, where all attempts to reasonably refine solvent molecules failed. Therefore, the SQUEEZE routine implemented in PLATON was applied and a squeezed electron count of 187 electrons per unit cell is obtained: == N: Total Potential Solvent Accessible Void Vol (SOLV-Map Value) ... 659 Ang^3 N: Electron Count Voids / Cell = 129 This is in accordance with two loosely packed iso-propanol molecules per asymmetric unit.

```
PLAT910_ALERT_3_G Missing # of FCF Reflection(s) Below Theta(Min).          1 Note
PLAT978_ALERT_2_G Number C-C Bonds with Positive Residual Density.         10 Info
PLAT992_ALERT_5_G Repd & Actual _reflns_number_gt Values Differ by          5 Check
```

```
   0 ALERT level A = Most likely a serious problem - resolve or explain
   1 ALERT level B = A potentially serious problem, consider carefully
   3 ALERT level C = Check. Ensure it is not caused by an omission or oversight
  20 ALERT level G = General information/check it is not something unexpected

   2 ALERT type 1 CIF construction/syntax error, inconsistent or missing data
   6 ALERT type 2 Indicator that the structure model may be wrong or deficient
   2 ALERT type 3 Indicator that the structure quality may be low
  12 ALERT type 4 Improvement, methodology, query or suggestion
   2 ALERT type 5 Informative message, check
```

It is advisable to attempt to resolve as many as possible of the alerts in all categories. Often the minor alerts point to easily fixed oversights, errors and omissions in your CIF or refinement strategy, so attention to these fine details can be worthwhile. In order to resolve some of the more serious problems it may be necessary to carry out additional measurements or structure refinements. However, the purpose of your study may justify the reported deviations and the more serious of these should normally be commented upon in the discussion or experimental section of a paper or in the "special_details" fields of the CIF. checkCIF was carefully designed to identify outliers and unusual parameters, but every test has its limitations and alerts that are not important in a particular case may appear. Conversely, the absence of alerts does not guarantee there are no aspects of the results needing attention. It is up to the individual to critically assess their own results and, if necessary, seek expert advice.

Publication of your CIF in IUCr journals

A basic structural check has been run on your CIF. These basic checks will be run on all CIFs submitted for publication in IUCr journals (*Acta Crystallographica*, *Journal of Applied Crystallography*, *Journal of Synchrotron Radiation*); however, if you intend to submit to *Acta Crystallographica Section C* or *E* or *IUCrData*, you should make sure that full publication checks are run on the final version of your CIF prior to submission.

Publication of your CIF in other journals

Please refer to the *Notes for Authors* of the relevant journal for any special instructions relating to CIF submission.

PLATON version of 13/07/2021; check.def file version of 13/07/2021

Datablock vale25 - ellipsoid plot

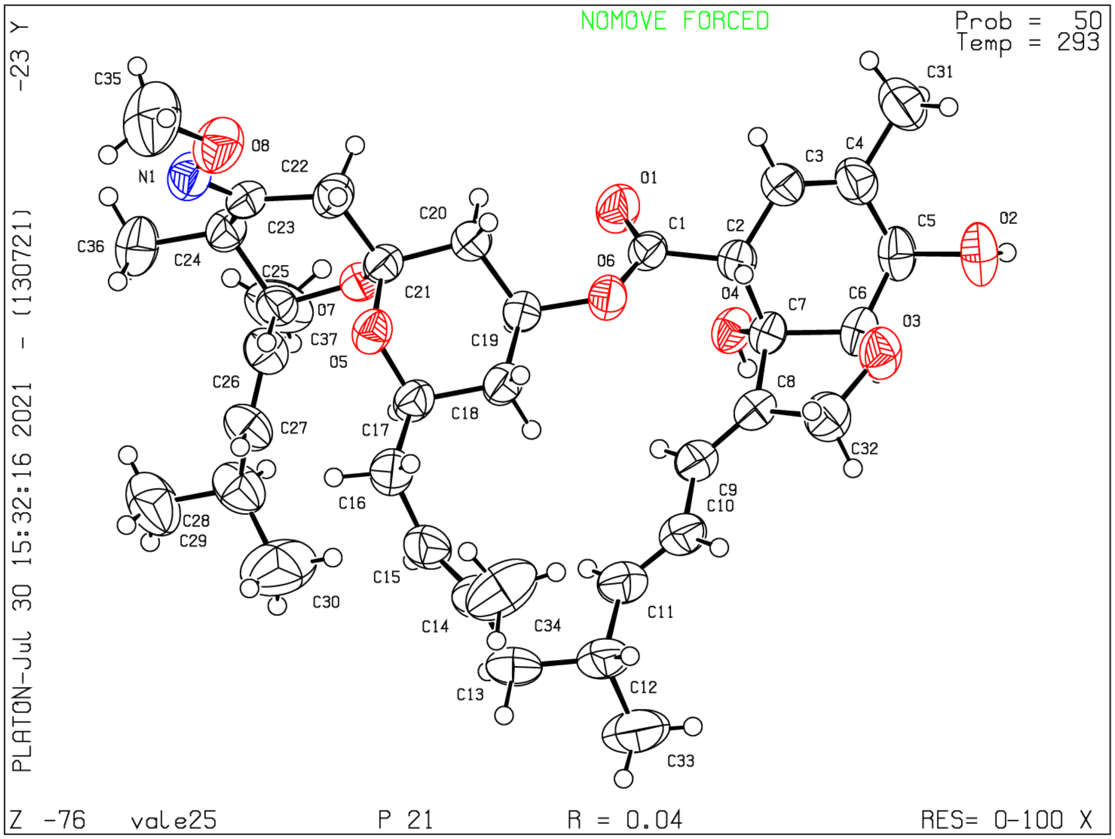

Supplement: Supplementary file 1 [file molecules-26-04869-s001.zip › molecules-1312668-supplementary/ESI/Moxi.2iPrOH_RT_checkcif.pdf]
