# Supplementary material for: Solvatomorphism of Moxidectin
Source: Molecules. 2021 Aug 11;26(16):4869. doi: 10.3390/molecules26164869 (PMC8398095; doi:10.3390/molecules26164869)
Supplement: Supplementary file 1 [file molecules-26-04869-s001.zip › molecules-1312668-supplementary/ESI/Moxidectin_esi.pdf]

Electronic Supporting Information (ESI)

Solvatomorphism of Moxidectin

Toni Grell[1], Mauro Barbero[2], Franco Pattarino,[2] Giovanni Battista Giovenzana[2] and Valentina Colombo[1*]

[1] Dipartimento di Chimica, Università degli Studi di Milano, Via Golgi 19, I-20133 Milano, Italy; valentina.colombo@unimi.it;
[2] Dipartimento di Scienze del Farmaco, Università del Piemonte Orientale A. Avogadro, L.go Donegani 2/3, I-28100 Novara, Italy;

* Correspondence: valentina.colombo@unimi.it; Tel.: +39 0250314450

1. Table of Contents

1. Table of Contents 1
2. Additional Information on X-Ray Diffraction 2
Variable-temperature X-ray powder diffraction (VT-XRPD) of amorphous moxidectin 2
Digitalized PXRD pattern for the solvatomorphs of moxidectin ..... **Error! Bookmark not defined.**
Comparison of the conformations of moxidectin in the different crystal structures ..... **Error! Bookmark not defined.**
Details on non-classical hydrogen bonding in the moxidectin single crystal structures 3
3. Additional Information on Quantum Chemical Calculations 4
Structural parameters of the calculated solvatomorph of *n*-butanol (Moxi·2*n*BuOH) 4
4. Literature 5

2. Additional Information on X-Ray Diffraction

Variable-temperature X-ray powder diffraction (VT-XRPD) of amorphous moxidectin

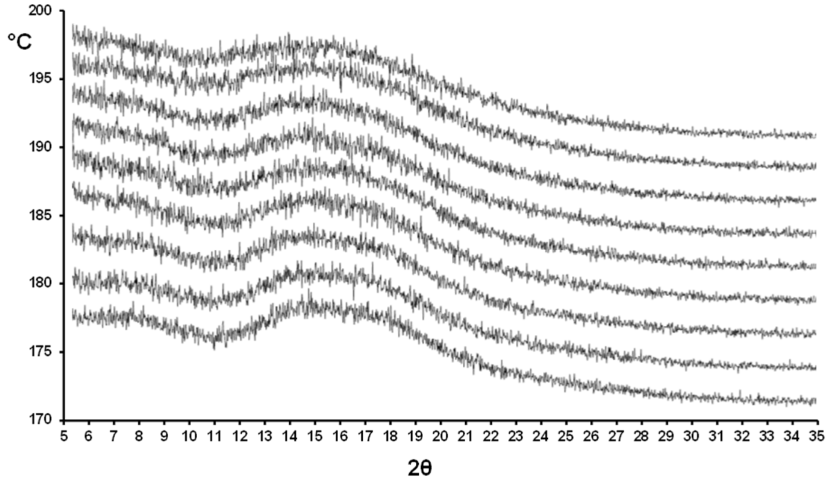

Figure S1. Variable-temperature X-ray powder diffraction (VT-XRPD) of amorphous moxidectin. Collection of diffractograms for of the amorphous form heated from 170 °C to 200 °C over the course of 5 hrs. This experiment demonstrates that no conversion of the amorphous to the crystalline form (“Form I”, Table 1) is taking place[1].

Details on hydrogen bonding in known moxidectin form

Table S1. Distances [Å] and type for strong and weak hydrogen bonds observed in form I and in the solvatomorphs discussed in this work.

Solvatomorph	N°	Interaction type O–H···O	O(–H)···O Distance	Type	N°	Interaction type C–H···O	C(–H)···O Distance
**Form I***	1	O4–H**···O1	2.74(1)	*Intra*mol. HB	1	C20–H18···O3	3.55(2)
					2	C36–H47···O5	3.46(2)
**Moxi·2EtOH**	1	O4–H53···O2’	2.760(2)	*Inter*mol. HB	1	C13–H10···O1	3.377(3)
	2	O2–H52···O10	2.700(3)	*Inter*mol. HB			
	3	O10–H65···O9	2.719(5)	*Inter*mol. HB			
	4	O9–H59···O4	2.782(4)	*Inter*mol. HB			
**Moxi·2*i*PrOH**	1	O4–H53···O2’	2.704(2)	*Inter*mol. HB	1	C13–H10···O1	3.429(3)
	2	O2–H52···O10	2.748(8)	*Inter*mol. HB			
	3	O10–H65···O9	2.784(8)	*Inter*mol. HB			
	4	O9–H59···O4	2.853(4)	*Inter*mol. HB			
**Moxi·1.5MeNO$_2$**	1	O4–H53···O1	2.763(5)	*Intra*mol. HB	1	C3–H2···O9	3.404(5)
	2	O2–H52···O3’	2.869(4)	*Inter*mol. HB	2	C20–H18···O9	3.495(5)
		O2’–H52’···O3	2.869(4)	*Inter*mol. HB	3	C20–H19···O10	3.254(6)
					4	C22–H21···O13	3.58(1)
					5	C29–H28···O14	3.54(2)
					6	C35–H44···O1	3.408(6)

***Moxidectin form I**, CSD reference code GETBOW[2]. **Sites for OH Hydrogen atoms were not assigned in the published structure[2].

3. Additional Information on Quantum Chemical Calculations

Structural parameters of the calculated solvatomorph of *n*-butanol (Moxi·2*n*BuOH)

The following lines contain the file content (text format) of the calculated structures in the *.cif format. The file can also be also found as supplementary material of this article.

```
data_Moxi.2nBuOH.theo
_symmetry_cell_setting          monoclinic
_symmetry_space_group_name_H-M   'P 21'
_symmetry_Int_Tables_number     4
_space_group_name_Hall          'P 21'
loop_
_symmetry_equiv_pos_site_id
_symmetry_equiv_pos_as_xyz
1 x,y,z
2 -x,1/2+y,-z
_cell_length_a                11.40648595
_cell_length_b                8.99960687
_cell_length_c                21.53422505
_cell_angle_alpha               90
_cell_angle_beta               93.779673
_cell_angle_gamma               90
_cell_volume                  2205.76
loop_
_atom_site_label
_atom_site_type_symbol
_atom_site_fract_x
_atom_site_fract_y
_atom_site_fract_z
O1 O 0.5413885344967 0.6674508484999 0.2763536883294
O3 O 0.6238686147472 0.7720905539405 0.5573083552618
O5 O 0.4022397552176 0.7971289716325 0.4977276192534
O7 O 0.4752869846368 0.5037568547674 0.3932005118957
O9 O 0.1844171384675 0.7396254164774 0.1090498444761
O11 O 0.3570470773777 0.7526335050569 0.2842739600892
O13 O 0.3304048028776 0.5604809185346 0.1017676552928
O15 O 0.3380462830003 0.95049214418799 -0.05091232970889
N21 N 0.3302222311775 0.7957566119048 -0.05988043983794
C23 C 0.4673391727433 0.7241196118789 0.3067334754946
C25 C 0.4836707824193 0.7679930791779 0.3745116433386
C27 C 0.6089025944754 0.8078656447164 0.3930131177031
C29 C 0.6616374512410 0.7848241944026 0.4493730141353
C31 C 0.6029405919529 0.6991531573512 0.4988612494977
C33 C 0.4725420658338 0.6707416532324 0.4844327526344
C35 C 0.4350278298434 0.6400867802370 0.4152222352539
C37 C 0.3031346826015 0.6564622919221 0.4182837165605
C39 C 0.2210082583492 0.5780873388784 0.3854012352946
C41 C 0.09516096296643 0.5977096374422 0.3882235774661
C43 C 0.01369639508846 0.5184501396116 0.3546661671156
C45 C -0.1169661052472 0.5406675036128 0.3545767809075
C47 C -0.1744452121647 0.5275303245427 0.2875075688313
C49 C -0.1246752741964 0.6351960681038 0.2422346656212
C51 C -0.06399865174010 0.5864448719498 0.1949597011569
C53 C -0.002612047540661 0.6776192533831 0.1483716013878
C55 C 0.1306097436100 0.6573778324886 0.1574733824101
C57 C 0.1823437546834 0.7100126995817 0.2207942059437
C59 C 0.3149284242201 0.6883991355325 0.2251116605578
C61 C 0.3702397610280 0.7627928328493 0.1704739189000
C63 C 0.3080316408720 0.7145213616435 0.1086130855247
C65 C 0.3526394314177 0.8019212267327 0.05402976936472
C67 C 0.3365026726107 0.7248508700604 -0.008216553865578
C69 C 0.3365519437947 0.5559108352572 -0.01021006628566
C71 C 0.2711380606279 0.4997910677786 0.04545264601659
C73 C 0.2635264113714 0.3346444331610 0.05527381003368
C75 C 0.1582212626910 0.2777463977742 0.06613872904903
C77 C 0.1272544364943 0.1218672981768 0.08392412899409
C79 C 0.07050550914453 0.03621658541466 0.02777116913418
C81 C 0.04475367229389 0.1277514195566 0.1376437083937
C83 C 0.7854357735123 0.8335128342725 0.4661632581181
C85 C 0.2862608610188 0.7694421218350 0.4689316524302
C87 C -0.1741299061060 0.4297751668367 0.3979797161602
C89 C -0.1482948094983 0.7960492157647 0.2558902609541
C91 C 0.3425144005436 1.02397197581030 -0.1094594348914
C93 C 0.2849687344143 0.4911678905078 -0.07179799115516
C95 C 0.3767899055183 0.2496008495901 0.05599670167153
H113 H 0.4288670001111 0.8660034227200 0.3801411293189
H115 H 0.6562633054358 0.8643915353594 0.3580024261505
H117 H 0.6437512513476 0.5888129781884 0.5017684235322
H119 H 0.4464569170826 0.5751307146249 0.5120194982740
H121 H 0.2504314268580 0.4953066934372 0.3532442742299
H123 H 0.06591811528557 0.6854561815183 0.4182435203247
H125 H 0.04284837277570 0.4311186995851 0.3243320185221
H127 H -0.1337462299023 0.6521790476080 0.3720151993937
H129 H -0.1628252506526 0.4135107108160 0.2714639992251
H131 H -0.2686177186346 0.5475904553743 0.2897461349071
H133 H -0.05333226150880 0.4669457734989 0.1901369173780
H135 H -0.02337502918201 0.7952817055046 0.1518589267491
H137 H -0.02982888713540 0.6429003152418 0.1008874613967
H139 H 0.1510815448961 0.5395709258954 0.1518428441307
H141 H 0.1625284344321 0.8279807065997 0.2260792309987
H143 H 0.1425796807763 0.6502453776943 0.2580162256221
H145 H 0.3371179291393 0.5706243068180 0.2264609502118
H147 H 0.4631580312460 0.7357443993624 0.1707155069883
H149 H 0.3611343715934 0.8832274915350 0.1744906007360
H151 H 0.3105606544588 0.91015022080795 0.05136228295073
```

H153 H 0.4468818827070 0.8214524414326 0.06380290186620
H155 H 0.4283235166323 0.5202653541421 -0.002104168110130
H157 H 0.1822758170055 0.5448174247679 0.04107708824974
H159 H 0.08333962299476 0.3529080764604 0.06275138768018
H161 H 0.2062998633330 0.06168102711254 0.1005175941403
H163 H 0.1325459081706 0.02533132204838 -0.008493713650742
H165 H -0.007376055656070 0.09515082762095 0.008333141487487
H167 H 0.04498388617891 -0.07507125961172 0.04178910146986
H169 H -0.03715010394398 0.1850334380749 0.1230390549380
H171 H 0.08581610389576 0.1871138437712 0.1777047489251
H173 H 0.02286850633335 0.01590327645455 0.1525521626375
H175 H 0.8409902102538 0.7385480190312 0.4803277679401
H177 H 0.7890196254762 0.9101378068100 2 0.5055390700776
H179 H 0.8227589690041 0.8871695930738 0.4267542746339
H181 H 0.2512992889772 0.8756271497873 0.4516365887045
H183 H 0.2284834622857 0.7271387884991 0.5035467809263
H185 H -0.2690463180184 0.4474224741107 0.3972628331082
H187 H -0.1370326870264 0.4432427061517 0.4457015815726
H189 H -0.1581910780086 0.3152719274527 0.3838249573555
H191 H -0.1250868431039 0.8712495259471 0.2190189255620
H193 H -0.09918829579209 0.8309441366331 0.2988456749052
H195 H -0.2415032209118 0.8108019328658 0.2631099230347
H197 H 0.3296274016783 1.1412978662237 -0.09959248579076
H199 H 0.2723832019362 0.98600491150080 -0.1424280763167
H201 H 0.4278808500470 1.008175803429533 -0.1284589777941
H203 H 0.3348788416158 0.5267196666709 -0.1105600303398
H205 H 0.1942921548443 0.5275628082659 -0.08126406031688
H207 H 0.2854920928737 0.3700479251046 -0.06950098071283
H209 H 0.4051776535875 0.2351134974467 0.008802548022165
H211 H 0.3693615917713 0.1391160616835 0.07582367638861
H213 H 0.4468315276466 0.3089020859818 0.08239854245363
H215 H 0.6382587465420 0.7008404248925 0.5919326833500
H217 H 0.4479898139918 0.4198141954125 0.4186325076219
O18 O 0.4483388791277 0.3388068277555 0.2863385583739
C98 C 0.5114382061588 0.3415186755610 0.2309366779656
C100 C 0.5257541317243 0.1846388398765 0.2059598909205
C102 C 0.5917427522007 0.07605078835898 0.2509154875156
C104 C 0.7104054843337 0.1353339368900 0.2774967742239
H220 H 0.5969115342626 0.3935374791787 0.2408361208749
H222 H 0.4628700493363 0.4087710426278 0.1956885165441
H224 H 0.5723982192448 0.1953523047609 0.1633879692902
H226 H 0.4387465008540 0.1394478180419 0.1921939160676
H228 H 0.4712524076485 0.4181790712031 0.3157283890754
H230 H 0.5369869006460 0.04918192070539 0.2890715964169
H232 H 0.6046614621774 -0.02830362157645 0.2261840049590
H234 H 0.6988969229992 0.2333754824438 0.3062091218679
H236 H 0.7570519855889 0.05214291359413 0.3065826233796
H238 H 0.7666699789670 0.1660043950046 0.2402982014716
O20 O 0.3532138171320 0.1132572461690 0.3389448420748
C106 C 0.2471524312898 0.09867736194877 0.3000856630261
C108 C 0.1519164861514 0.02490631934046 0.3352948608926
C110 C 0.1199367731792 0.1126536855956 0.3927585197328
C112 C 0.01095304550464 0.05173656098846 0.4214003878310
H240 H 0.2647030993111 0.03255349229308 0.2591078362838
H242 H 0.2178170152442 0.2090325787731 0.2839803058732
H244 H 0.1798938646177 -0.08770048512857 0.3483152043165
H246 H 0.07391649177507 0.01322584835843 0.3031223944373
H248 H 0.4018553227230 0.1884575956259 0.3186464675450
H250 H 0.1059031647114 0.2294535227523 0.3803618398186
H252 H 0.1939724564394 0.1116820167030 0.4274337414670
H254 H 0.02257050610941 -0.06488798055921 0.4345797219335
H256 H -0.009506531027469 0.1133132515966 0.4630626435058
H258 H -0.06565777043474 0.05848294360946 0.3882111276840

#END

4. Literature

[1] US2013143956-A1, WO2013082373-A1, **2013**.

[2] N. E. Beddall, P. D. Howes, M. V.J. Ramsay, S. M. Roberts, A. M.Z. Slawin, D. R. Sutherland, E. P. Tiley, D. J. Williams, *Tetrahedron Lett.* **1988**, *29*, 2595.
