# Supplementary material for: Solvatomorphism of Moxidectin
Source: Molecules. 2021 Aug 11;26(16):4869. doi: 10.3390/molecules26164869 (PMC8398095; doi:10.3390/molecules26164869)

checkCIF/PLATON report

Structure factors have been supplied for datablock(s) vale53

THIS REPORT IS FOR GUIDANCE ONLY. IF USED AS PART OF A REVIEW PROCEDURE FOR PUBLICATION, IT SHOULD NOT REPLACE THE EXPERTISE OF AN EXPERIENCED CRYSTALLOGRAPHIC REFEREE.

No syntax errors found. CIF dictionary Interpreting this report

Datablock: vale53

```
Bond precision:  C-C = 0.0036 A          Wavelength=0.71073

Cell:            a=11.1913(4)     b=8.8440(3)        c=21.9594(8)
                 alpha=90         beta=93.747(3)     gamma=90
Temperature:     120 K

                Calculated                 Reported
Volume          2168.80(13)                2168.80(13)
Space group     P 21                       P 21
Hall group      P 2yb                      P 2yb
Moiety formula  C37 H53 N O8, 2(C2 H6 O)  C37 H53 N O8, 2(C2 H6 O)
Sum formula     C41 H65 N O10              C41 H65 N O10
Mr              731.94                     731.94
Dx,g cm-3       1.121                      1.121
Z               2                          2
Mu (mm-1)       0.079                      0.079
F000            796.0                      796.0
F000'           796.40
h,k,lmax        16,13,32                   16,13,32
Nref            14520[ 7679]               13011
Tmin,Tmax       0.989,0.992                0.963,1.000
Tmin'           0.988

Correction method= # Reported T Limits: Tmin=0.963 Tmax=1.000
AbsCorr = MULTI-SCAN

Data completeness= 1.69/0.90      Theta(max)= 31.571

R(reflections)= 0.0525( 10522)    wR2(reflections)= 0.1254( 13011)

S = 1.013                 Npar= 485
```

The following ALERTS were generated. Each ALERT has the format
test-name_ALERT_alert-type_alert-level.
Click on the hyperlinks for more details of the test.

Alert level C

DIFMX02_ALERT_1_C The maximum difference density is > 0.1*ZMAX*0.75
The relevant atom site should be identified.

Author Response: The highest electron density peak is located close to the oxygen O9 and thus a split disorder is suggested. However, all attempts to mod- el this disorder failed. If refined to a split position, the bond pa- rameters of the second site do not refine to be chemically reasona- ble. One O9 site will give a bond length (1.398 A) as well as a C-C-O bond angle (107) in the expected range whereas both parameters are too large for the second site (1.457 A and 120 ; 30% occupancy). While the bond length can be adjusted using SADI or SAME, the bond angle remains too large. (2) If refined isotropic the displacement parameters for both sites are acceptable. However, this causes large Q peaks in between the sites. Instead, when refining both sites ani- sotropically, the ADP for the second position (the same that causes problems with the bond angle) yields a deformed ADP for which SHELXL once again suggests a split refinement. It is therefore concluded, that the residual electron density is caused by an artefact due to the inferior data quality The description as atom with a slightly large ADP is therefore more accurate.

PLAT097_ALERT_2_C Large Reported Max. (Positive) Residual Density 0.65 eA-3

Author Response: The highest electron density peak is located close to the oxygen O9 and thus a split disorder is suggested. However, all attempts to mod- el this disorder failed. If refined to a split position, the bond pa- rameters of the second site do not refine to be chemically reasona- ble. One O9 site will give a bond length (1.398 A) as well as a C-C-O bond angle (107) in the expected range whereas both parameters are too large for the second site (1.457 A and 120 ; 30% occupancy). While the bond length can be adjusted using SADI or SAME, the bond angle remains too large. (2) If refined isotropic the displacement parameters for both sites are acceptable. However, this causes large Q peaks in between the sites. Instead, when refining both sites ani- sotropically, the ADP for the second position (the same that causes problems with the bond angle) yields a deformed ADP for which SHELXL once again suggests a split refinement. It is therefore concluded, that the residual electron density is caused by an artefact due to the inferior data quality The description as atom with a slightly large ADP is therefore more accurate.

```
PLAT244_ALERT_4_C Low    'Solvent' Ueq as Compared to Neighbors of        C38 Check
```

Alert level G

```
PLAT002_ALERT_2_G Number of Distance or Angle Restraints on AtSite          4 Note
PLAT003_ALERT_2_G Number of Uiso or Uij Restrained non-H Atoms ...          1 Report
PLAT007_ALERT_5_G Number of Unrefined Donor-H Atoms .............          2 Report
PLAT172_ALERT_4_G The CIF-Embedded .res File Contains DFIX Records          1 Report
PLAT186_ALERT_4_G The CIF-Embedded .res File Contains ISOR Records          1 Report
PLAT395_ALERT_2_G Deviating  X-O-Y    Angle From 120 for O8             108.9 Degree
PLAT398_ALERT_2_G Deviating  C-O-C    Angle From 120 for O3             105.9 Degree
PLAT791_ALERT_4_G Model has Chirality at C2         (Sohnke SpGr)           R Verify
```

Author Response: The absolute structure of the molecule is known and was not deter- mined by anomalous-dispersion effects. The sample was a weak anomalous scatterer. The absolute structure parameter was calculated to be -0.3(3) which is meaningless for the determination of the absolute structure. It was thus deleted from the *.cif file.

```
PLAT791_ALERT_4_G Model has Chirality at C5         (Sohnke SpGr)           R Verify
```

Author Response: The absolute structure of the molecule is known and was not deter- mined by anomalous-dispersion effects. The sample was a weak anomalous scatterer. The absolute structure parameter was calculated to be -0.3(3) which is meaningless for the determination of the absolute structure. It was thus deleted from the *.cif file.

```
PLAT791_ALERT_4_G Model has Chirality at C6         (Sohnke SpGr)           R Verify
```

Author Response: The absolute structure of the molecule is known and was not deter- mined by anomalous-dispersion effects. The sample was a weak anomalous scatterer. The absolute structure parameter was calculated to be -0.3(3) which is meaningless for the determination of the absolute structure. It was thus deleted from the *.cif file.

```
PLAT791_ALERT_4_G Model has Chirality at C7         (Sohnke SpGr)           S Verify
```

Author Response: The absolute structure of the molecule is known and was not deter- mined by anomalous-dispersion effects. The sample was a weak anomalous scatterer. The absolute structure parameter was calculated to be -0.3(3) which is meaningless for the determination of the absolute structure. It was thus deleted from the *.cif file.

```
PLAT791_ALERT_4_G Model has Chirality at C12        (Sohnke SpGr)           R Verify
```

Author Response: The absolute structure of the molecule is known and was not deter- mined by anomalous-dispersion effects. The sample was a weak anomalous scatterer. The absolute structure parameter was calculated to be -0.3(3) which is meaningless for the determination of the absolute structure. It was thus deleted from the *.cif file.

```
PLAT791_ALERT_4_G Model has Chirality at C17         (Sohnke SpGr)          R Verify
```

Author Response: The absolute structure of the molecule is known and was not deter- mined by anomalous-dispersion effects. The sample was a weak anomalous scatterer. The absolute structure parameter was calculated to be -0.3(3) which is meaningless for the determination of the absolute structure. It was thus deleted from the *.cif file.

```
PLAT791_ALERT_4_G Model has Chirality at C19         (Sohnke SpGr)          S Verify
```

Author Response: The absolute structure of the molecule is known and was not deter- mined by anomalous-dispersion effects. The sample was a weak anomalous scatterer. The absolute structure parameter was calculated to be -0.3(3) which is meaningless for the determination of the absolute structure. It was thus deleted from the *.cif file.

```
PLAT791_ALERT_4_G Model has Chirality at C21         (Sohnke SpGr)          R Verify
```

Author Response: The absolute structure of the molecule is known and was not deter- mined by anomalous-dispersion effects. The sample was a weak anomalous scatterer. The absolute structure parameter was calculated to be -0.3(3) which is meaningless for the determination of the absolute structure. It was thus deleted from the *.cif file.

```
PLAT791_ALERT_4_G Model has Chirality at C24         (Sohnke SpGr)          S Verify
```

Author Response: The absolute structure of the molecule is known and was not deter- mined by anomalous-dispersion effects. The sample was a weak anomalous scatterer. The absolute structure parameter was calculated to be -0.3(3) which is meaningless for the determination of the absolute structure. It was thus deleted from the *.cif file.

```
PLAT791_ALERT_4_G Model has Chirality at C25         (Sohnke SpGr)          S Verify
```

Author Response: The absolute structure of the molecule is known and was not deter- mined by anomalous-dispersion effects. The sample was a weak anomalous scatterer. The absolute structure parameter was calculated to be -0.3(3) which is meaningless for the determination of the absolute structure. It was thus deleted from the *.cif file.

```
PLAT860_ALERT_3_G Number of Least-Squares Restraints .............          9 Note
PLAT910_ALERT_3_G Missing # of FCF Reflection(s) Below Theta(Min).          1 Note
PLAT912_ALERT_4_G Missing # of FCF Reflections Above STh/L=  0.600        579 Note
PLAT941_ALERT_3_G Average HKL Measurement Multiplicity ...........        3.6 Low
PLAT978_ALERT_2_G Number C-C Bonds with Positive Residual Density.         19 Info
```

```
   0 ALERT level A = Most likely a serious problem - resolve or explain
   0 ALERT level B = A potentially serious problem, consider carefully
   3 ALERT level C = Check. Ensure it is not caused by an omission or oversight
  22 ALERT level G = General information/check it is not something unexpected
```

```
 1 ALERT type 1 CIF construction/syntax error, inconsistent or missing data
 6 ALERT type 2 Indicator that the structure model may be wrong or deficient
 3 ALERT type 3 Indicator that the structure quality may be low
14 ALERT type 4 Improvement, methodology, query or suggestion
 1 ALERT type 5 Informative message, check
```

It is advisable to attempt to resolve as many as possible of the alerts in all categories. Often the minor alerts point to easily fixed oversights, errors and omissions in your CIF or refinement strategy, so attention to these fine details can be worthwhile. In order to resolve some of the more serious problems it may be necessary to carry out additional measurements or structure refinements. However, the purpose of your study may justify the reported deviations and the more serious of these should normally be commented upon in the discussion or experimental section of a paper or in the "special_details" fields of the CIF. checkCIF was carefully designed to identify outliers and unusual parameters, but every test has its limitations and alerts that are not important in a particular case may appear. Conversely, the absence of alerts does not guarantee there are no aspects of the results needing attention. It is up to the individual to critically assess their own results and, if necessary, seek expert advice.

Publication of your CIF in IUCr journals

A basic structural check has been run on your CIF. These basic checks will be run on all CIFs submitted for publication in IUCr journals (*Acta Crystallographica*, *Journal of Applied Crystallography*, *Journal of Synchrotron Radiation*); however, if you intend to submit to *Acta Crystallographica Section C* or *E* or *IUCrData*, you should make sure that full publication checks are run on the final version of your CIF prior to submission.

Publication of your CIF in other journals

Please refer to the *Notes for Authors* of the relevant journal for any special instructions relating to CIF submission.

PLATON version of 13/07/2021; check.def file version of 13/07/2021

Datablock vale53 - ellipsoid plot

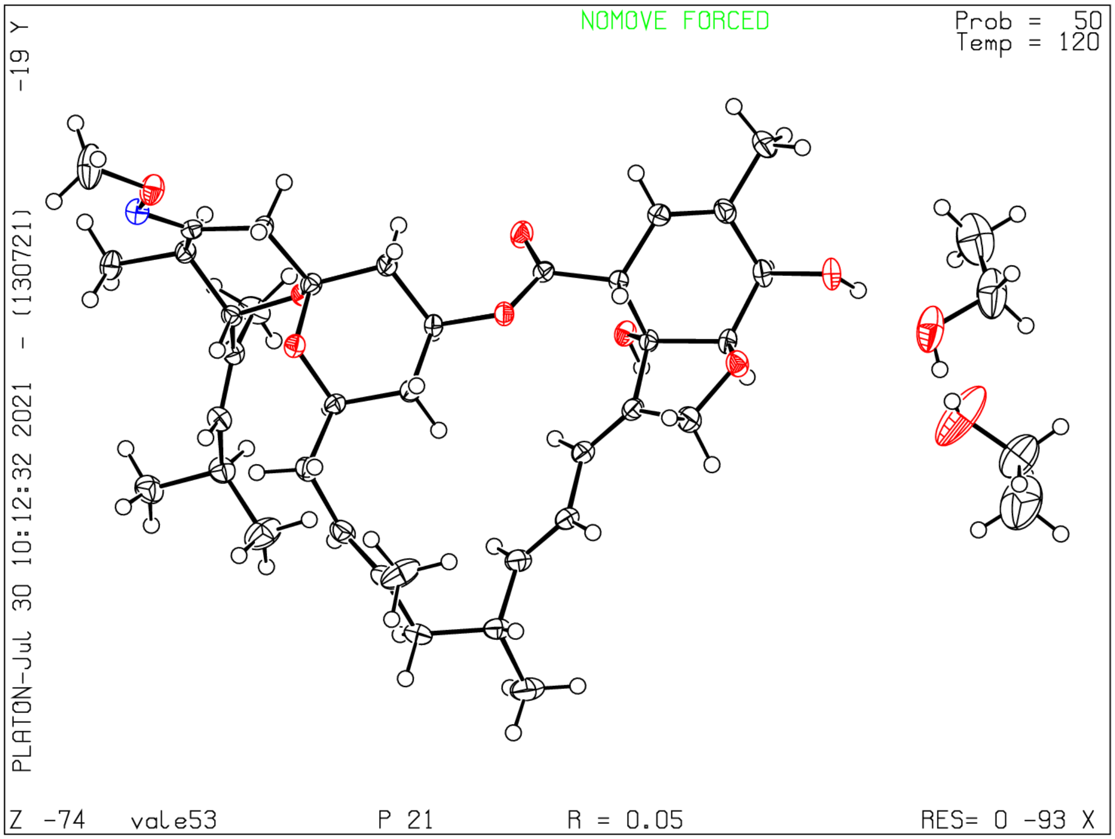

Supplement: Supplementary file 1 [file Moxi.2EtOH_checkcif.pdf › molecules-1312668-supplementary/ESI/Moxi.2EtOH_checkcif.pdf]
