# Supplementary material for: Solvatomorphism of Moxidectin
Source: Molecules. 2021 Aug 11;26(16):4869. doi: 10.3390/molecules26164869 (PMC8398095; doi:10.3390/molecules26164869)

checkCIF/PLATON report

Structure factors have been supplied for datablock(s) vale66

THIS REPORT IS FOR GUIDANCE ONLY. IF USED AS PART OF A REVIEW PROCEDURE FOR PUBLICATION, IT SHOULD NOT REPLACE THE EXPERTISE OF AN EXPERIENCED CRYSTALLOGRAPHIC REFEREE.

No syntax errors found. CIF dictionary Interpreting this report

Datablock: vale66

```
Bond precision:  C-C = 0.0029 A          Wavelength=0.71073

Cell:            a=11.4417(3)     b=8.9507(2)        c=21.7210(6)
                 alpha=90         beta=92.301(3)     gamma=90
Temperature:     120 K

                Calculated                 Reported
Volume          2222.68(10)                2222.68(10)
Space group     P 21                       P 21
Hall group      P 2yb                      P 2yb
Moiety formula  C37 H53 N O8, 2(C3 H8 O)  C37 H53 N O8, 2(C3 H8 O)
Sum formula     C43 H69 N O10              C43 H69 N O10
Mr              759.99                     759.99
Dx,g cm-3       1.136                      1.136
Z               2                          2
Mu (mm-1)       0.079                      0.079
F000            828.0                      828.0
F000'           828.41
h,k,lmax        16,13,32                   16,13,31
Nref            15081[ 7965]               13370
Tmin,Tmax       0.991,0.992                0.906,1.000
Tmin'           0.984

Correction method= # Reported T Limits: Tmin=0.906 Tmax=1.000
AbsCorr = MULTI-SCAN

Data completeness= 1.68/0.89      Theta(max)= 31.719

R(reflections)= 0.0448( 11518)    wR2(reflections)= 0.1169( 13370)

S = 1.021                  Npar= 538
```

The following ALERTS were generated. Each ALERT has the format
test-name_ALERT_alert-type_alert-level.
Click on the hyperlinks for more details of the test.

Alert level C

```
PLAT202_ALERT_3_C Isotropic non-H Atoms in Anion/Solvent .........          1 Check
              C40
```

Author Response: The position of one solvent molecule was poorly defined and one atom could only be refined isotropically.

```
PLAT220_ALERT_2_C NonSolvent   Resd 1  C   Ueq(max)/Ueq(min) Range        3.4 Ratio
```

Alert level G

```
PLAT002_ALERT_2_G Number of Distance or Angle Restraints on AtSite         12 Note
PLAT007_ALERT_5_G Number of Unrefined Donor-H Atoms ..............          6 Report
PLAT172_ALERT_4_G The CIF-Embedded .res File Contains DFIX Records          2 Report
PLAT302_ALERT_4_G Anion/Solvent/Minor-Residue Disorder (Resd  2  )       100% Note
PLAT302_ALERT_4_G Anion/Solvent/Minor-Residue Disorder (Resd  3  )       100% Note
PLAT302_ALERT_4_G Anion/Solvent/Minor-Residue Disorder (Resd  4  )       100% Note
PLAT302_ALERT_4_G Anion/Solvent/Minor-Residue Disorder (Resd  5  )       100% Note
PLAT304_ALERT_4_G Non-Integer Number of Atoms in ..... (Resd  2  )       7.79 Check
PLAT304_ALERT_4_G Non-Integer Number of Atoms in ..... (Resd  3  )       7.79 Check
PLAT304_ALERT_4_G Non-Integer Number of Atoms in ..... (Resd  4  )       4.21 Check
PLAT304_ALERT_4_G Non-Integer Number of Atoms in ..... (Resd  5  )       4.21 Check
PLAT395_ALERT_2_G Deviating  X-O-Y    Angle From 120 for O8             108.4 Degree
PLAT398_ALERT_2_G Deviating  C-O-C    Angle From 120 for O3             106.0 Degree
PLAT417_ALERT_2_G Short Inter D-H..H-D       H52      ..H10B     .       1.94 Ang.
                                                       x,y,z  =      1_555 Check
PLAT791_ALERT_4_G Model has Chirality at C2          (Sohnke SpGr)          R Verify
```

Author Response: The absolute structure of the molecule is known and was not deter- mined by anomalous-dispersion effects. The sample was a weak anoma- lous scatterer. The absolute structure parameter was calculated to be -0.2(2) which is meaningless for the determination of the absolute structure. It was thus deleted from the *.cif file.

```
PLAT791_ALERT_4_G Model has Chirality at C5          (Sohnke SpGr)          R Verify
```

Author Response: The absolute structure of the molecule is known and was not deter- mined by anomalous-dispersion effects. The sample was a weak anoma- lous scatterer. The absolute structure parameter was calculated to be -0.2(2) which is meaningless for the determination of the absolute structure. It was thus deleted from the *.cif file.

```
PLAT791_ALERT_4_G Model has Chirality at C6          (Sohnke SpGr)          R Verify
```

Author Response: The absolute structure of the molecule is known and was not deter- mined by anomalous-dispersion effects. The sample was a weak anomalous scatterer. The absolute structure parameter was calculated to be -0.2(2) which is meaningless for the determination of the absolute structure. It was thus deleted from the *.cif file.

```
PLAT791_ALERT_4_G Model has Chirality at C7          (Sohnke SpGr)          S Verify
```

Author Response: The absolute structure of the molecule is known and was not deter- mined by anomalous-dispersion effects. The sample was a weak anomalous scatterer. The absolute structure parameter was calculated to be -0.2(2) which is meaningless for the determination of the absolute structure. It was thus deleted from the *.cif file.

```
PLAT791_ALERT_4_G Model has Chirality at C12         (Sohnke SpGr)          R Verify
```

Author Response: The absolute structure of the molecule is known and was not deter- mined by anomalous-dispersion effects. The sample was a weak anomalous scatterer. The absolute structure parameter was calculated to be -0.2(2) which is meaningless for the determination of the absolute structure. It was thus deleted from the *.cif file.

```
PLAT791_ALERT_4_G Model has Chirality at C17         (Sohnke SpGr)          R Verify
```

Author Response: The absolute structure of the molecule is known and was not deter- mined by anomalous-dispersion effects. The sample was a weak anomalous scatterer. The absolute structure parameter was calculated to be -0.2(2) which is meaningless for the determination of the absolute structure. It was thus deleted from the *.cif file.

```
PLAT791_ALERT_4_G Model has Chirality at C19         (Sohnke SpGr)          S Verify
```

Author Response: The absolute structure of the molecule is known and was not deter- mined by anomalous-dispersion effects. The sample was a weak anomalous scatterer. The absolute structure parameter was calculated to be -0.2(2) which is meaningless for the determination of the absolute structure. It was thus deleted from the *.cif file.

```
PLAT791_ALERT_4_G Model has Chirality at C21         (Sohnke SpGr)          R Verify
```

Author Response: The absolute structure of the molecule is known and was not deter- mined by anomalous-dispersion effects. The sample was a weak anomalous scatterer. The absolute structure parameter was calculated to be -0.2(2) which is meaningless for the determination of the absolute structure. It was thus deleted from the *.cif file.

```
PLAT791_ALERT_4_G Model has Chirality at C24         (Sohnke SpGr)          S Verify
```

Author Response: The absolute structure of the molecule is known and was not deter- mined by anomalous-dispersion effects. The sample was a weak anomalous scatterer. The absolute structure parameter was calculated to be -0.2(2) which is meaningless for the determination of the absolute structure. It was thus deleted from the *.cif file.

```
PLAT791_ALERT_4_G Model has Chirality at C25         (Sohnke SpGr)          S Verify
```

Author Response: The absolute structure of the molecule is known and was not deter- mined by anomalous-dispersion effects. The sample was a weak anomalous scatterer. The absolute structure parameter was calculated to be -0.2(2) which is meaningless for the determination of the absolute structure. It was thus deleted from the *.cif file.

```
PLAT860_ALERT_3_G Number of Least-Squares Restraints .............          9 Note
PLAT910_ALERT_3_G Missing # of FCF Reflection(s) Below Theta(Min).          1 Note
PLAT912_ALERT_4_G Missing # of FCF Reflections Above STh/L=  0.600        592 Note
PLAT941_ALERT_3_G Average HKL Measurement Multiplicity ...........        3.6 Low
PLAT978_ALERT_2_G Number C-C Bonds with Positive Residual Density.         15 Info
```

```
   0 ALERT level A = Most likely a serious problem - resolve or explain
   0 ALERT level B = A potentially serious problem, consider carefully
   2 ALERT level C = Check. Ensure it is not caused by an omission or oversight
  29 ALERT level G = General information/check it is not something unexpected

   0 ALERT type 1 CIF construction/syntax error, inconsistent or missing data
   6 ALERT type 2 Indicator that the structure model may be wrong or deficient
   4 ALERT type 3 Indicator that the structure quality may be low
  20 ALERT type 4 Improvement, methodology, query or suggestion
   1 ALERT type 5 Informative message, check
```

It is advisable to attempt to resolve as many as possible of the alerts in all categories. Often the minor alerts point to easily fixed oversights, errors and omissions in your CIF or refinement strategy, so attention to these fine details can be worthwhile. In order to resolve some of the more serious problems it may be necessary to carry out additional measurements or structure refinements. However, the purpose of your study may justify the reported deviations and the more serious of these should normally be commented upon in the discussion or experimental section of a paper or in the "special_details" fields of the CIF. checkCIF was carefully designed to identify outliers and unusual parameters, but every test has its limitations and alerts that are not important in a particular case may appear. Conversely, the absence of alerts does not guarantee there are no aspects of the results needing attention. It is up to the individual to critically assess their own results and, if necessary, seek expert advice.

Publication of your CIF in IUCr journals

A basic structural check has been run on your CIF. These basic checks will be run on all CIFs submitted for publication in IUCr journals (*Acta Crystallographica*, *Journal of Applied Crystallography*, *Journal of Synchrotron Radiation*); however, if you intend to submit to *Acta Crystallographica Section C* or *E* or *IUCrData*, you should make sure that full publication checks are run on the final version of your CIF prior to submission.

Publication of your CIF in other journals

Please refer to the *Notes for Authors* of the relevant journal for any special instructions relating to CIF submission.

PLATON version of 13/07/2021; check.def file version of 13/07/2021

Datablock vale66 - ellipsoid plot

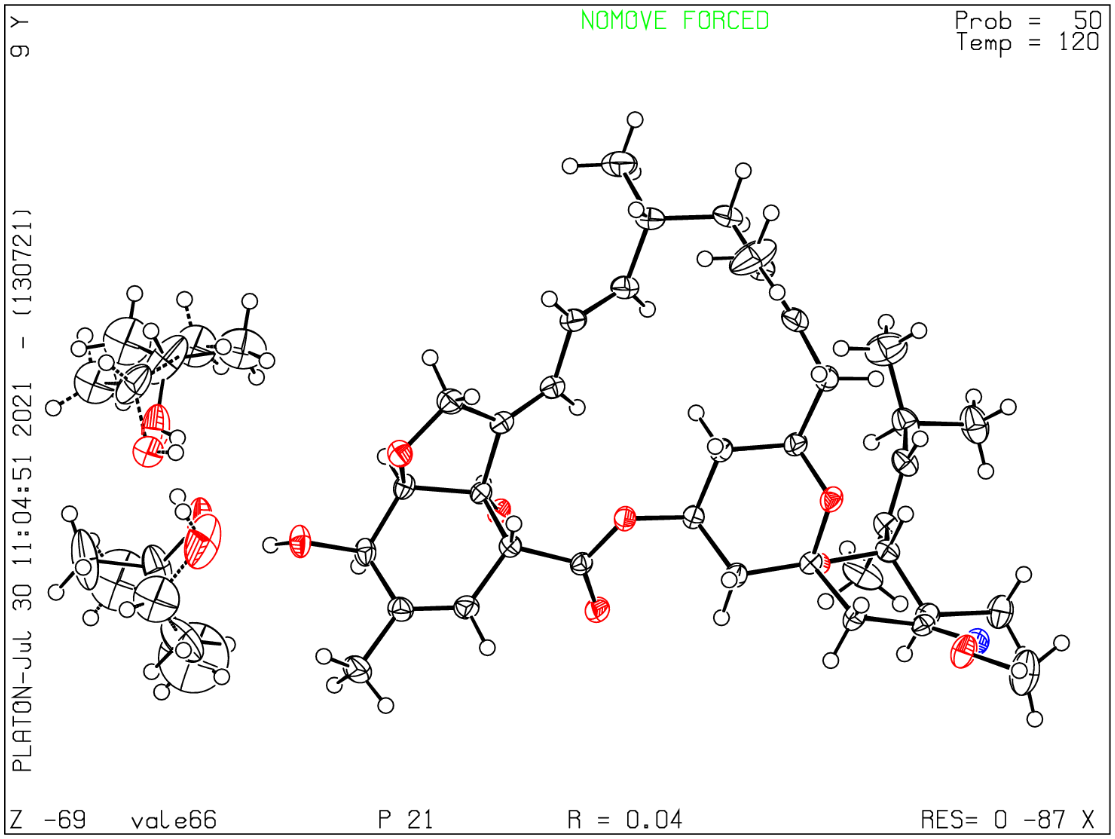

Supplement: Supplementary file 1 [file Moxi.2iPrOH_checkcif.pdf › molecules-1312668-supplementary/ESI/Moxi.2iPrOH_checkcif.pdf]
